# Hepatitis C in HIV-infected individuals: a systematic review and meta-analysis of estimated prevalence in Africa

**DOI:** 10.7448/IAS.19.1.20711

**Published:** 2016-06-09

**Authors:** Tiago Castro Lopes Azevedo, Marcel Zwahlen, Andri Rauch, Matthias Egger, Gilles Wandeler

**Affiliations:** 1 Department of Infectious Diseases, Bern University Hospital, University of Bern, Switzerland; 2 Institute of Social and Preventive Medicine (ISPM), University of Bern, Bern, Switzerland; 3 Centre for Infectious Disease Epidemiology and Research (CIDER), University of Cape Town, South Africa; 4 Department of Infectious Diseases, University of Dakar, Dakar, Senegal

**Keywords:** HIV infection, hepatitis C infection, Africa, meta-analysis, antibody test, polymerase chain reaction, genotype

## Abstract

**Introduction:**

Although hepatitis C virus (HCV) screening is recommended for all HIV-infected patients initiating antiretroviral therapy, data on epidemiologic characteristics of HCV infection in resource-limited settings are scarce.

**Methods:**

We searched PubMed and EMBASE for studies assessing the prevalence of HCV infection among HIV-infected individuals in Africa and extracted data on laboratory methods used. Prevalence estimates from individual studies were combined for each country using random-effects meta-analysis. The importance of study design, population and setting as well as type of test (anti-HCV antibody tests and polymerase chain reactions) was examined with meta-regression.

**Results:**

Three randomized controlled trials, 28 cohort studies and 121 cross-sectional analyses with 108,180 HIV-infected individuals from 35 countries were included. The majority of data came from outpatient populations (55%), followed by blood donors (15%) and pregnant women (14%). Based on estimates from 159 study populations, anti-HCV positivity prevalence ranged between 3.3% (95% confidence interval (CI) 1.8–4.7) in Southern Africa and 42.3% (95% CI 4.1–80.5) in North Africa. Study design, type of setting and age distribution did not influence this prevalence significantly. The prevalence of replicating HCV infection, estimated from data of 29 cohorts, was 2.0% (95% CI 1.5–2.6). Ten studies from nine countries reported the HCV genotype of 74 samples, 53% were genotype 1, 24% genotype 2, 14% genotype 4 and 9% genotypes 3, 5 or 6.

**Conclusions:**

The prevalence of anti-HCV antibodies is high in HIV-infected patients in Africa, but replicating HCV infection is rare and varies widely across countries.

## Introduction

Worldwide, four to five million HIV-infected individuals have concomitant chronic hepatitis C virus (HCV) infection [1]. HIV accelerates the progression of liver injury due to HCV, including liver cirrhosis and hepatocellular carcinoma (HCC) [2]. In the era of highly active antiretroviral therapy (ART), HCV infection has become a major cause of death in HIV-infected individuals in high-income countries [3,4]. The World Health Organization (WHO) recommends that all HIV-infected individuals be screened for HCV infection, ideally at presentation or before the initiation of ART [5]. These guidelines are based on a wealth of data from high-income countries, showing that HIV-infected patients, especially persons who inject drugs (PWID) and men who have sex with men (MSM), are at increased risk of HCV infection [6,7].

In resource-limited settings, data on the prevalence of HCV infection in HIV-infected populations and its impact on long-term clinical outcomes are limited. In particular, prevalence estimates of HCV co-infection in African HIV programmes vary widely across settings and are mainly based on studies that used serological tests with low specificity [8]. For instance, of 500 HIV-infected patients investigated for HCV infection in Rakai, Uganda, 31 (6.2%) had a positive HCV-antibody serology but no replicative HCV infection was found when using polymerase chain reaction (PCR) [9]. Similar discrepancies between HCV serology and PCR results were found in pregnant women in Southern Africa and outpatients in West Africa [10,11].

In settings with limited resources for health, HIV treatment and care programmes must give priority to cost-effective interventions. A thorough assessment of the prevalence of HIV/HCV co-infection, including chronic active HCV infection and the genotypes involved, is needed to guide screening and treatment strategies [12]. We performed a systematic review of the literature and a meta-analysis to synthesize the prevalence of HCV-antibody positivity and model the prevalence of replicating HCV infection in HIV-infected individuals in Africa.

## Methods

A protocol for this systematic review was written and registered with the International prospective register of systematic reviews (PROSPERO registration number CRD42015016355) [13]. The reporting of the review followed the PRISMA guidelines [14].

### Search strategy and study selection

We searched PubMed and EMBASE on 3 March 2016 for studies assessing the prevalence of HCV-co-infection in HIV-infected individuals in Africa. In PubMed, we combined free text words and medical subject headings (MESH) describing the study population and the outcome (see Supplementary file). The PubMed search was adapted for EMBASE. We considered any type of study including randomized controlled trials (RCTs), cohort studies and cross-sectional analyses that included at least 20 HIV-infected patients. No language restrictions were applied. We excluded studies that did not describe the study population or the HCV tests used. If studies reported estimates of the prevalence of HIV/HCV co-infection for different population groups, each estimate was included separately. If several articles reported on the same population, we included the report with the most detailed description of the study population and type of diagnostic test used. Two reviewers (TC and GW) independently selected studies first based on titles and abstracts, and, in a second step, based on the full text of potentially eligible articles.

### Data extraction

Two independent reviewers (TC and GW) extracted data on the study design, setting (urban, rural or both), type of institution (teaching hospital, other hospital, health centre, community, laboratory, prison), age categories (adults over 15 years, children, both), study population (outpatient, inpatient, blood donors, pregnant women, community sample, special populations) as well as on the characteristics of the HCV test performed (type of test, description and commercial name). HCV prevalence from serological tests, rapid point-of-care tests, immunoblots and PCR was considered. Furthermore, data on the risk factors associated with HCV infection and HCV genotypes were also extracted if available. Discrepancies in data extracted were resolved through discussions with a third reviewer (M.E.).

### Ethical considerations

As this study was a meta-analysis of published studies and did not include the collection of individual patient data, no specific ethics approval was needed.

### Statistical analysis

We used the total number of tested persons and the number of positive results (serology or PCR) to calculate the prevalence estimate and exact 95% confidence intervals (95% CI). We derived study weights from the width of the 95% CI. We combined estimates of the prevalence of HIV/HCV co-infection by country using random-effects meta-analysis. We analyzed the prevalence of co-infection stratified by the type of test used: anti-HCV antibody tests (standard serological tests and rapid tests) and PCR. Countries were classified into low (<1.5%), moderate (1.5–3.5%) and high (>3.5%) prevalence, according to published definitions [15]. In order to explain the potential heterogeneity obtained by pooling prevalence estimates, we used random-effects meta-regression to assess the association between HCV prevalence and several study characteristics, including the type of study, year of publication, setting, population and age. Variables associated with anti-HCV antibody prevalence (*p*<0.1) were included into the multivariable model.

Serology and PCR data on the prevalence of HIV/HCV co-infection within the same study were available only for few studies and countries. We used Poisson regression with logarithmic link to estimate the ratio of prevalence estimates based on serology and prevalence based on PCR from studies with available data for both tests [16]. Analyses were conducted separately by region (North Africa, West Africa, Central Africa, East Africa and Southern Africa) and the region-specific correction factor was applied to the country-level estimates of serology-based prevalence to estimate the prevalence of replicating HCV co-infection for countries without PCR results. All statistical analyses were performed using Stata software version 12.1 (College Station, TX).

## Results

### Study and participant characteristics

**Figure 1:**
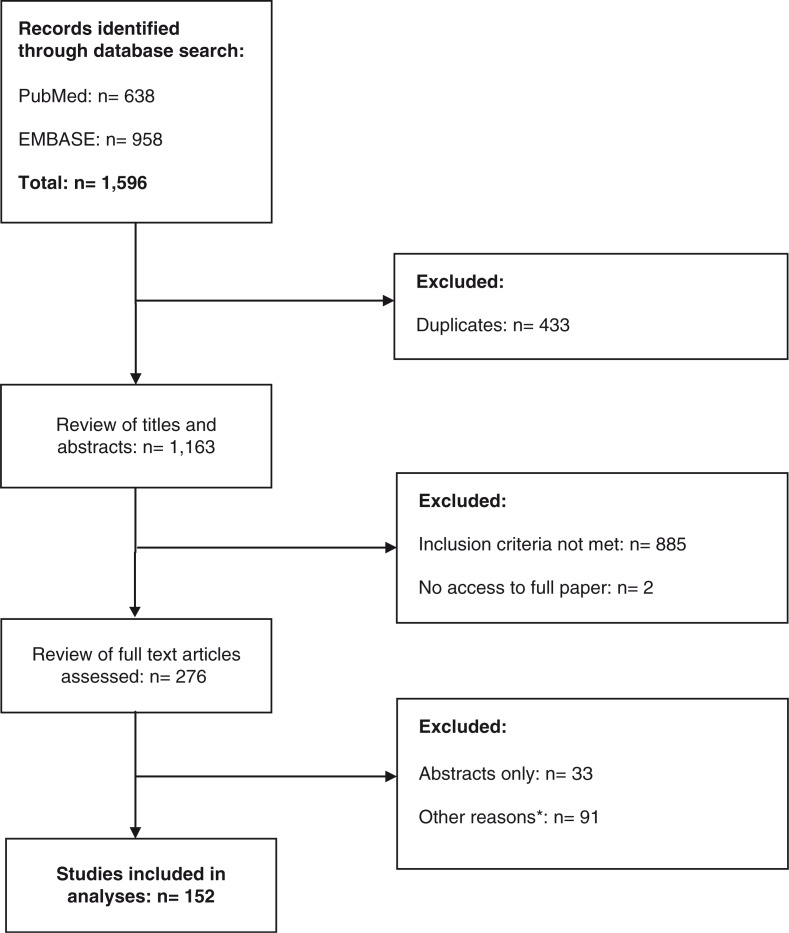
Flow Chart of identification of eligible studies (last search conducted on 3.3.16). *Other reasons were abstracts without published full manuscripts (*n*=33), no original studies (25), no specific estimates on HIV/HCV-coinfection (33), less than 20 HIV-infected patients (16), insufficient information on HCV testing or study population (8), no reported estimates from the African continent (3), data based on a population with liver disease only (1), reported data on patients included in another study (5).

The PubMed and EMBASE searches identified 1163 publications. The screening of titles and abstracts resulted in the selection of 276 potentially eligible studies, for which the full-text articles were evaluated. Of these, 152 publications, including three RCTs, 28 cohort studies and 121 cross-sectional analyses met our inclusion criteria (Figure 1).

Six studies reported HCV prevalence estimates from two or more populations. A detailed description of study characteristics by publication and country is given in Supplementary Table 1. The studies originated from 35 African countries. The number of studies per country ranged from one (Angola, Burundi, Central African Republic, Equatorial Guinea, Gabon, Guinea-Bissau, Lesotho, Namibia, Niger, Sudan, Tchad and Zimbabwe) to 37 (Nigeria). Analyses for West Africa were based on 65 studies, followed by East Africa (42), Southern Africa (26), Central Africa (19) and North Africa (7) (Table 1). In total, 24 studies (16%) included data from rural settings and 36 (24%) from paediatric populations. The majority of studies (84; 55%) were based on outpatients, followed by blood donors (23; 15%) and pregnant women (22; 14%). Eleven studies reported data from special populations, including prisoners in Libya and Nigeria; PWID in Kenya, Libya, Senegal and Tanzania; MSM in Tanzania and the Republic of South Africa (RSA); female sex workers in Ethiopia and the Democratic Republic of Congo (DRC) and forensic cases in RSA (Supplementary Table 2).

**Table 1:** Study characteristics by region

	Total (%)	North Africa	West Africa	Central Africa	East Africa	Southern Africa
Number of study populations	159	7	65	19	42	26
Study design						
RCT	4 (2.5)	0	1	1	1	1
Cohort	29 (18.2)	0	11	6	6	6
Cross-sectional	126 (79.3)	7	53	12	35	19
Year of publication						
Before 2000	14 (8.8)	1	4	3	2	4
2000–2009	63 (39.6)	1	27	6	20	9
After 2009	82 (51.6)	5	34	10	20	13
Study population						
Outpatients	84 (52.9)	4	36	11	19	14
Inpatients	11 (6.9)	0	2	0	5	4
Blood donors	23 (14.5)	0	12	3	5	3
Pregnant women	22 (13.8)	0	11	2	6	3
Community	8 (5.0)	1	2	2	3	0
Special populations^a^	11 (6.9)	2	2	1	4	2
Age category						
Adults	116 (76.3)	7	49	15	25	20
Children	9 (5.9)	0	3	1	5	0
Both	27 (18.8)	0	11	3	9	4
Setting						
Urban	120 (83.3)	6	50	11	36	17
Rural	11 (7.7)	0	1	1	3	6
Both	13 (9.0)	0	8	3	2	0
Type of test						
Serology	101 (54.0)	6	43	10	23	18
Rapid test	28 (15.0)	0	14	2	11	1
Immuno-blot	29 (15.5)	1	8	8	5	7
PCR	29 (15.5)	2	11	4	6	6

a Two studies in prisoners in Libya and Nigeria, four studies in people who inject drugs (PWID) in Kenya, Libya, Senegal and Tanzania, one study in men who have sex with men (MSM) and PWID in Tanzania, one study in MSM in the Republic of South Africa (RSA), two studies in female sex workers in Ethiopia and the Democratic Republic of Congo and one study in forensic cases in RSA. (See Supplementary Table 2 for details.)

### Estimates of HIV/HCV co-infection prevalence from different tests

In total, 187 HCV prevalence estimates were available, including 158 from anti-HCV antibody tests. Among these, 101 were available from serology, including enzyme-linked immunosorbent assay (ELISA) and enzyme immunoassay (EIA), and 28 from rapid point-of-care tests. Although 29 studies confirmed serological test results with immunoblot assays, full data from both test types were only available for 10 of these. Twenty-nine studies showed PCR test results. The number of estimates from HCV antibody tests by country ranged from 1 to 35 (Nigeria). Cameroon and Nigeria were the only countries to have PCR results from three different studies. Median HCV viral load from PCR was reported in five studies and ranged from 11,969 IU/mL in Morocco to 2,735,000 IU/mL in Burkina Faso. In total, 18 studies from 14 countries (Morocco, Burkina Faso, The Gambia, Ivory Coast, Nigeria, Cameroon, Gabon, Guinea-Bissau, Ethiopia, Uganda, Lesotho, Malawi, RSA and Zambia) reported HCV prevalence estimates from HCV-antibody screening tests and PCR confirmation within the same study population (Supplementary Table 3).

### Prevalence of hepatitis C in HIV-infected patients, by region and country

**Figure 2:**
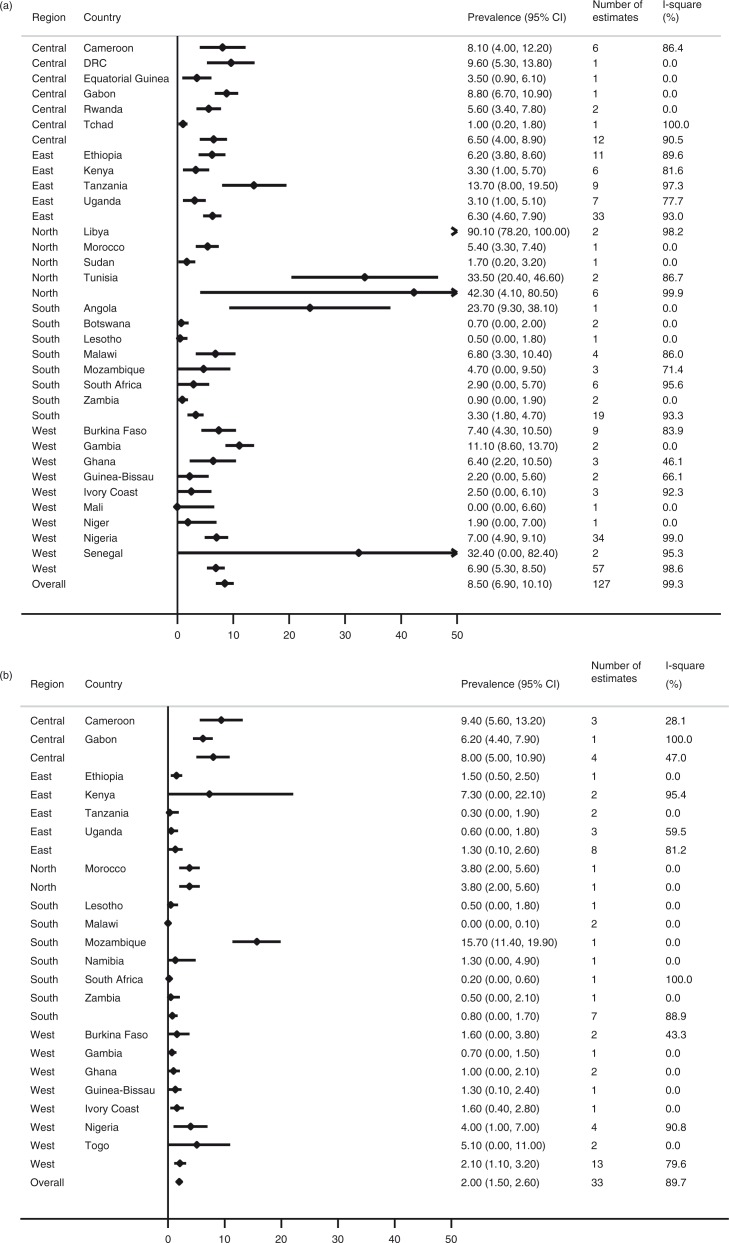
Meta-analysis of hepatitis C prevalence among HIV-infected individuals, by country, according to anti-HCV antibody tests (a) and PCR (b) results.

The prevalence of HIV/HCV infection according to HCV antibody screening tests (serology and rapid tests only) was 8.5% (95% CI 6.9–10.1) (Figure 2a). The prevalence of anti-HCV antibody positivity was highest in North Africa (42.3%, 95% CI 4.1–80.5), followed by West Africa (6.9%, 95% CI 5.3–8.5), Central Africa (6.5%, 95% CI 4.0–8.9), East Africa (6.3%, 95% CI 4.6–7.9) and southern Africa (3.3%, 95% CI 1.8–4.7). Libya (90.1%), Tunisia (33.5%), Senegal (32.4%) and Angola (23.7%) had the highest prevalence of anti-HCV positive HIV-infected patients. For Libya, Tunisia and Senegal, the result relied on studies in prisoners or in cohorts with high proportions of PWID. The study from Angola included only 38 HIV-infected patients and drug use was not assessed.

The overall estimate for confirmed, replicating HCV infection was 2.0% (95% CI 1.5–2.6) (Figure 2b). The prevalence of HCV PCR-positivity was highest in Central Africa (8.0%, 95% CI 5.0–10.9), followed by North Africa (3.8%, 95% CI 2.0–5.6), West Africa (2.1%, 95% CI 1.1–3.2), East Africa (1.3%, 95% CI 0.1–2.6) and southern Africa (0.8%, 95% CI 0–1.7). Mozambique (15.7%) and Cameroon (9.4%) had the highest PCR-positivity prevalence. The estimate from Mozambique relied on a single report, including 300 HIV-infected individuals in an urban outpatient clinic where no injection drug use (IDU) was reported. In Cameroon, three studies confirmed the high prevalence of replicating HCV infections with minimal heterogeneity between studies (I-squared: 28.1%, *p*=0.25).

There was little evidence that the prevalence of HCV-antibody positivity depended on the type of setting (*p*=0.79), age group (*p*=0.56), study design (*p*=0.35) or year of publication (*p*=0.29) (Supplementary Table 4). The prevalence estimates were higher in the group of studies performed in special populations, which included analyses in prisoners and PWID (Supplementary Table 2). However, when the nine studies in special populations were excluded from the meta-regression, the type of population did not influence the overall estimates significantly (*p*=0.22). Only one study from special populations reported estimates from PCR results (PWID in Kenya). In meta-analyses restricted to outpatient populations (*n*=70 studies), HCV prevalence was similar to the overall estimates (Supplementary Figure 1).

**Figure 3:**
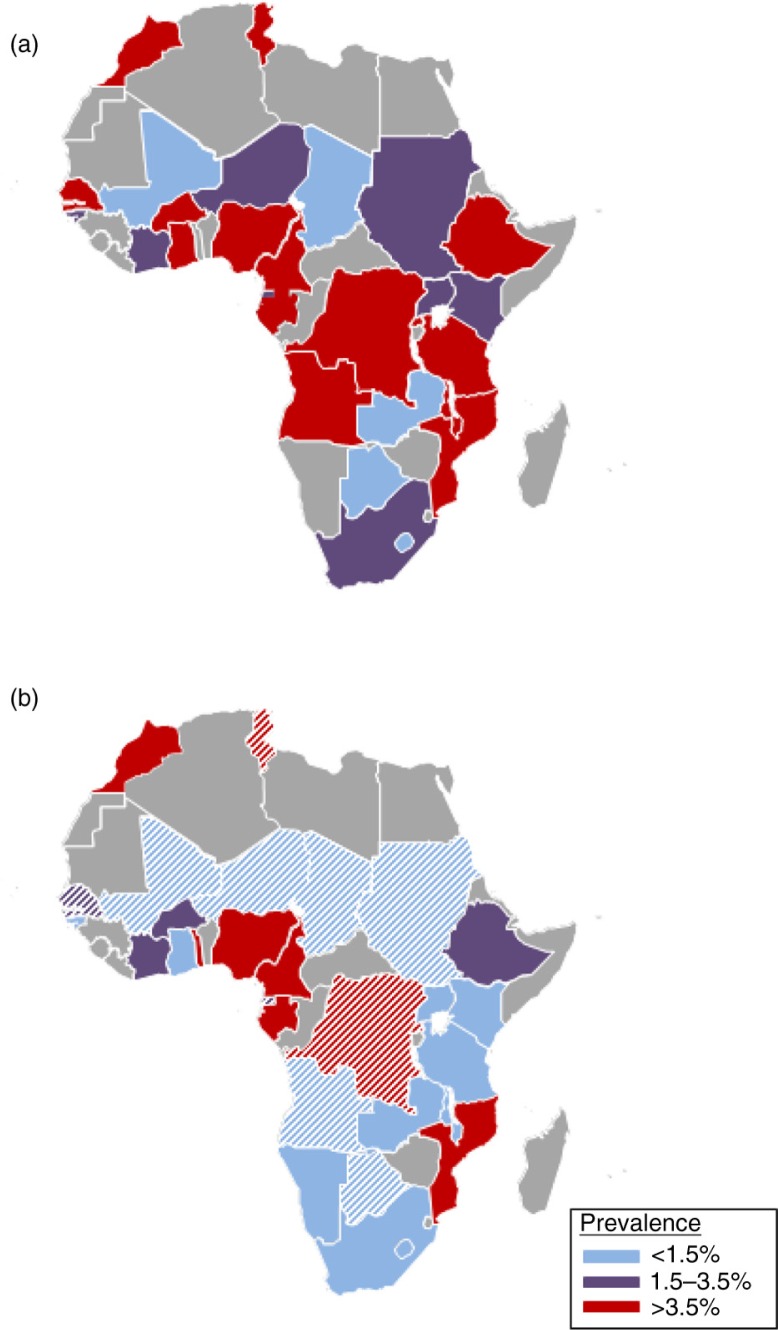
Prevalence of hepatitis C among HIV-infected populations from (a) antibody tests (excluding immunoblots), (b) and PCR tests. **Combined estimates from random-effect meta-analysis (solid fill) and imputed estimates from meta-regression (striped fill). Studies in special populations were excluded for this analysis**.

Figure 3 shows the comparison between HCV prevalence estimates from screening tests and PCR, by country, after the exclusion of studies from special populations. The majority of countries (16/29) were classified into the high-prevalence group, according to HCV screening tests, whereas only five of them (Botswana, Lesotho, Mali, Tchad and Zambia) were included in the low-prevalence group (Figure 3a). However, of 20 countries with available data, only five (Cameroon, Gabon, Morocco, Mozambique, Nigeria and Togo) remained in the high-prevalence group in the analysis based on PCR-positivity (Figure 3b). Of note, the estimates for replicating HCV infection prevalence in Morocco, Togo and Mozambique each relied on a single, small study. When the region-specific correction factor (Supplementary Table 3) was applied to countries for which only serological data were available, imputed PCR prevalence estimates were obtained for 11 additional countries (Figure 3 and Supplementary Table 5).

###  HCV genotypes

**Figure 4:**
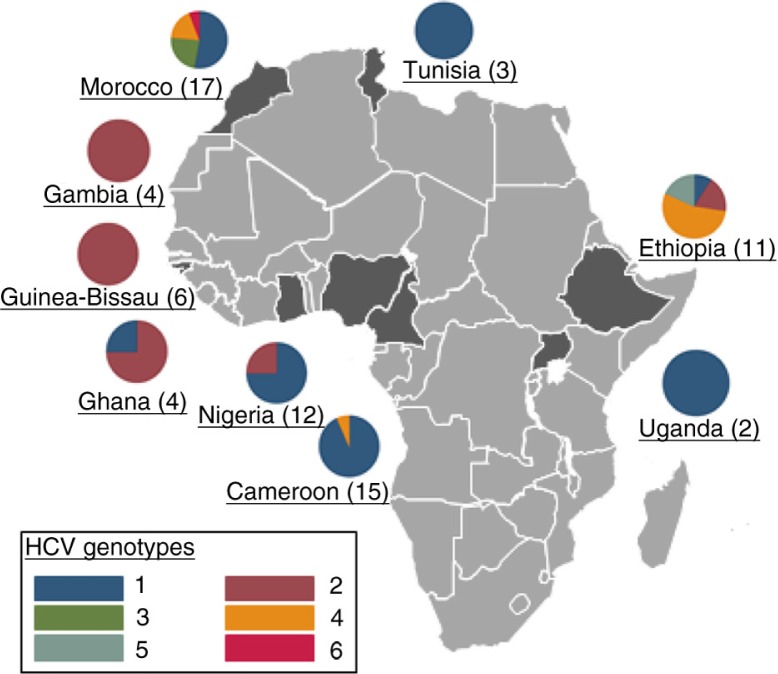
HCV genotype distribution in nine countries with available data. **The numbers in brackets are the number of samples evaluated**.

Ten studies reported data on HCV genotypes. Nine of these were from outpatient clinics in Cameroon, Ethiopia, Gambia, Ghana, Guinea-Bissau, Nigeria, Morocco and Tunisia and one from inpatients in Uganda. Of 74 samples analyzed, 39 (53%) were of genotype 1, 18 (24%) of genotype 2 and 10 (14%) genotype 4. Among the studies with available data, genotypes 3 (four samples) and 6 (one sample) were only described in Morocco, and genotype 5 (two samples) in Ethiopia. Figure 4 shows the genotype distribution for each country with available data. Cameroon was the only country for which two studies could be included. Genotype 1 was the most common one in all countries, except for Gambia, Ghana and Guinea-Bissau, where genotype 2 was most prevalent, and Ethiopia, where genotype 4 was most prevalent.

## Discussion

Our meta-analysis aimed at assessing the prevalence of hepatitis C in HIV-infected patients in Africa and describing the differences in estimates between diagnostic tests. Overall, 152 studies, including 159 separate study populations across 35 countries, were included. We found a prevalence of anti-HCV antibodies of 8.5%, with large differences across study populations and countries. The prevalence of hepatitis C among HIV-infected patients according to anti-HCV screening tests was over four times higher than the estimates obtained for replicating HCV infection, which was 2%. Only 19% of the studies included reported data on PCR-confirmed HCV infections and 10 studies showed HCV genotype distributions.

The overall anti-HCV antibody prevalence obtained in our meta-analysis was higher than the estimates from the general population, which ranged from 2.0% in East Africa to 2.8% in West Africa according to a recent meta-analysis [15]. Globally, the high HCV prevalence in HIV-infected individuals is driven by IDU and sexual transmission in MSM. In our analysis, only two studies focused on MSM and five on PWID, whereas IDU was only reported in eight studies, including three from North Africa, two from Tanzania, one from Kenya, one from Senegal and one from Nigeria. As a consequence, it was very difficult to evaluate the impact of MSM or IDU behaviour on the HCV seroprevalence from our results. Although HIV epidemics in PWID have been well-described in North Africa [17], data on PWID in sub-Saharan (SSA) are scarce [6,18]. Nevertheless, it is considered that IDU is on the rise in SSA, especially in large urban settings, and could contribute to the spread of HCV infection [19]. Despite the emergence of HCV infection epidemics in HIV-infected MSM in industrialized settings [7,20], no study has reported an increase in sexual transmission of HCV in HIV-infected MSM in Africa to date. In meta-regression analyses, we found no association between the main types of study populations (including outpatients, inpatients, blood donors and pregnant women) and anti-HCV prevalence.

The prevalence of HIV/HCV-coinfection was generally much lower when only PCR-confirmed estimates were considered. There are several potential explanations for the large differences between HIV/HCV-coinfection prevalence estimates obtained from anti-HCV antibody and PCR tests. Between 15 and 40% of HCV infections resolve without treatment, depending on HIV status, sex, host genetic determinants as well as HCV genotype [21,22]. However, as spontaneous clearance of HCV infections cannot explain the four-fold difference between the prevalence of anti-HCV positivity and replicating infections found in our study, false-positive anti-HCV antibody results seem to play a major role, as recognized previously [8]. Although most studies report having used anti-HCV screening tests according to the manufacturer's instructions, the possibility remains that defective test kits were used in some studies. Alternatively, the presence of false-positive anti-HCV serologies has been linked to cross-reactions with other types of antibodies. The polyclonal B-cell activation following certain bacterial, parasitic and viral infections typically produces antibodies that are not specific to one type of infection [23]. In addition, cross-reactions between these immune reagents and host antigens also occur. Thus, immune responses to other infections as well as the stimulation of auto-antibodies could explain false-positive anti-HCV test results in certain settings. For instance, past schistosomal infection, which is very common in many parts of SSA, has been associated with false-positive anti-HCV results in Egypt and Uganda [9,24]. Interestingly, among the countries with available data from HCV antibody tests and PCR in our study, Cameroon and Morocco, which have low schistosoma infection prevalence [25], seemed to have the lowest proportion of false-positive anti-HCV tests.

The evaluation of HCV genotypes is central in understanding the natural history, transmission chains and evolution of HCV infection. Despite the very low number of samples assessed for HCV genotypes in the studies included in our meta-analysis, the results seem to be in agreement with those from HIV-uninfected cohorts reviewed in a recent meta-analysis [26]. Gower *et al*. showed that HCV genotype 4 predominated in East and Central Africa, whereas genotypes 1 and 2 were most prevalent in West Africa. Among the seven countries with data in the Gowan *et al*. study as well as in ours, genotype 1 predominated in Tunisia, Morocco and Nigeria in both meta-analyses; genotype 2 was present in the majority of samples from the Gambia, Ghana and Guinea-Bissau and genotype 4 was the most common one in Ethiopia. However, the comparison of our results with the study by Gowan *et al*. is limited by their exclusion of studies with less than 1000 patients and the low number of genotypes assessed in our study.

We reviewed an extensive body of literature without any restriction on calendar period, language or age category, in order to perform a comprehensive assessment of HCV prevalence in HIV-infected individuals in Africa. In order to compare HCV prevalence estimates from different diagnostic tests, we extracted detailed data on each test assessed in every study. The most important limitation of our systematic review was the paucity of data available from many African countries and the limited number of patients included in a large proportion of studies. For instance, 79 (50%) HIV/HCV prevalence estimates relied on study populations of less than 200 HIV-infected individuals. Only 12 (34%) countries had more than two studies included in the analyses, limiting the generalizability of the HCV seroprevalence estimates in the majority of countries. Furthermore, only a limited number of studies confirmed their HCV antibody test results with PCR and, in those that did, estimates for the different tests performed were often not reported. Although very informative, our modelling of PCR-positivity prevalence estimates does not replace original data for those countries in which no original data were available. Finally, data on HCV genotypes reported in this systematic review cannot be used to infer general trends for specific countries or regions due to the very limited number of samples assessed.

## Conclusions

Our results show that despite the high HCV seroprevalence in HIV-infected individuals in Africa, the prevalence of viremic HCV infections and genotype distributions seem to be comparable to those from published data of the general population. Our study underlines the potential over-estimation in HCV-infection prevalence reported by many studies of HIV-infected patients in settings endemic for other infections such as schistosomiasis. In order to understand the differences in the prevalence of replicating HCV infections across regions and countries, future studies need to correlate the data from PCR results with detailed assessments of risk factors for HCV infection, including IDU and unsterile medical injections. Ways to overcome the challenges posed by sub-optimal screening tests and expensive PCR methods should be studied, including the implementation of HCV testing using dried blood spots as well as novel diagnostic methods such as hepatitis C core antigen tests [27] and HCV antibody rapid test with better specificity [28].

## Supplementary Material

Hepatitis C in HIV-infected individuals: a systematic review and meta-analysis of estimated prevalence in Africa
