# Supplementary material for: Hepatitis C in HIV-infected individuals: a systematic review and meta-analysis of estimated prevalence in Africa
Source: J Int AIDS Soc. 2016 Jun 9;19(1):20711. doi: 10.7448/IAS.19.1.20711 (PMC4904089; doi:10.7448/IAS.19.1.20711)
Supplement: Hepatitis C in HIV-infected individuals: a systematic review and meta-analysis of estimated prevalence in Africa [file JIAS-19-20711-s001.pdf]

APPENDIX

A1. The search Strategy for PubMed

(((((HIV Infections[MeSH] OR HIV[MeSH] OR hiv[tiab] OR hiv-1*[tiab] OR hiv-2*[tiab] OR hiv1[tiab] OR hiv2[tiab] OR hiv infect*[tiab] OR human immunodeficiency virus[tiab] OR human immunedeficiency virus[tiab] OR human immuno-deficiency virus[tiab] OR human immune-deficiency virus[tiab] OR ((human immun*[tiab]) AND (deficiency virus[tiab])) OR acquired immunodeficiency syndrome[tiab] OR acquired immunedeficiency syndrome[tiab] OR acquired immuno-deficiency syndrome[tiab] OR acquired immunedeficiency syndrome[tiab] OR ((acquired immun*[tiab]) AND (deficiency syndrome[tiab])) OR "sexually transmitted diseases, Viral"[MeSH:noexp]))) AND (((("Hepatitis C"[Mesh]) OR hepatitis c[Title/Abstract])) OR hcv[Title/Abstract]))) AND (((((africa[MeSH Terms]) OR africa[Text Word]))) OR ((((developing countries[MeSH Terms]) OR ((low income countries OR middle income countries OR "low and middle income countries" OR resource-limited settings OR resource-poor settings OR low-resource settings))))))

A2. Supplementary tables

Supplementary table 1: Characteristics of studies

Study	First author	Year	Design	Setting	Age Category	Population	HIV-infected	Rapid test Positive	Serology Positive	Immunoblot Positive	PCR Positive
Angola[1]	Guimarães	2013	CS	urban	adult	outpatient	38	-	9	-	-
Botswana 1[2]	Patel	2011	CS	urban	adult	outpatient	252	-	2	-	-
Botswana 2[3]	Wester	2006	CS	urban	adult	outpatient	160	-	0	-	-
Burkina Faso 1[4]	Collenberg	2006	CO	both	both	pregnant	33	-	0	0	-
Burkina Faso 2[5]	Kania	2009	CS	urban	adult	blood donors	49	-	3	-	-
Burkina Faso 3[6]	Nagalo	2011	CS	both	adult	blood donors	100	-	17	-	-
Burkina Faso 4[7]	Nagalo	2012	CS	both	adult	blood donors	433	-	35	-	-
Burkina Faso 5[8]	Simpore	2004	CS	urban	both	pregnant	108	-	12	-	-
Burkina Faso 6[9]	Simpore	2005	CS	urban	adult	pregnant	58	-	9	7	0
Burkina Faso 7[10]	Simpore	2006	CS	urban	adult	pregnant	207	10	-	-	-
Burkina Faso 8[11]	Zeba	2011	CS	urban	adult	pregnant	378	9	-	-	9
Burkina Faso 9[12]	Kirakoya	2014	CS	unknown	adult	blood donors	761	73	-	-	-
Burundi[13]	Ntakarutimana	1995	CS	both	both	outpatient	151	-	-	15	-
Cameroon 1a[14]	Feldt	2013	CO	unknown	adult	outpatient	279	-	7	-	-
Cameroon 1b[14]	Feldt	2013	CO	unknown	paediatric	outpatient	200	-	5	-	-
Cameroon 2[15]	Laurent	2007	CS	rural	adult	community	35	-	-	3	-
Cameroon 3[16]	Laurent	2010	CO	urban	adult	outpatient	169	-	28	-	21
Cameroon 4[17]	Mbanya	2003	CS	urban	adult	blood donors	20	-	-	2	-
Cameroon 5[18]	Ndjomou	2002	CS	urban	both	outpatient	124	-	-	13	8
Cameroon 6[19]	Nkengasong	1994	CS	unknown	adult	outpatient	80	-	14	12	8
Cameroon 7[20]	Noubiap	2013	CS	unknown	adult	blood donors	22	3	-	-	-
Cameroon 8[21]	Noubiap	2015	CO	both	both	community	531	-	38	-	-
Centr. Afr. Rep.[22]	Pawlotsky	1995	CS	urban	adult	outpatient	30	-	-	1	-
DRC 1[23]	Kabinda	2010	CO	urban	adult	outpatient	209	-	20	-	-
DRC 2a[24]	Laurent	2001	CS	urban	adult	special	390	-	-	29	-
DRC 2b[24]	Laurent	2001	CS	urban	adult	pregnant	31	-	-	2	-

Study	First author	Year	Design	Setting	Age Category	Population	HIV-infected	Rapid test Positive	Serology Positive	Immunoblot Positive	PCR Positive
Equatorial Guinea 1[25]	Xie	2015	CS	both	adult	blood donors	230	8	-	-	-
Ethiopia 1[26]	Abreha	2011	CS	urban	adult	outpatient	734	-	50	-	11
Ethiopia 2a[27]	Ayele	2002	CS	urban	adult	community	165	-	-	8	-
Ethiopia 2b[27]	Ayele	2002	CS	urban	adult	pregnant	309	-	-	9	-
Ethiopia 2c[27]	Ayele	2002	CS	urban	adult	special	150	-	-	8	-
Ethiopia 3[28]	Diro	2008	CS	urban	adult	blood donors	27	-	6	-	-
Ethiopia 4[29]	Tessema	2010	CS	urban	adult	blood donors	239	-	4	-	-
Ethiopia 5[30]	Tiruneh	2008	CS	urban	adult	pregnant	57	1	-	-	-
Ethiopia 6[31]	Balew	2014	CS	both	both	inpatient	395	5	-	-	-
Ethiopia 7[32]	Hadush	2013	CS	urban	adult	outpatient	174	16	-	-	-
Ethiopia 8[33]	Manyazewal	2014	CS	urban	adult	outpatient	500	18	-	-	-
Ethiopia 9[34]	Taye	2013	CO	urban	adult	outpatient	387	25	-	-	-
Ethiopia 10[35]	Wondimeneh	2013	CS	urban	adult	outpatient	400	22	-	-	-
Ethiopia 11[36]	Abera	2014	CS	urban	paediatric	outpatient	253	-	14	-	-
Ethiopia 12[37]	Mulu	2013	CS	urban	adult	outpatient	269	-	59	-	-
Gabon 1[38]	Rouet	2015	CS	urban	adult	outpatient	762	-	67	-	47
Gambia 1[39]	Jobarteh	2010	CS	both	adult	outpatient	572	-	63	7	4
Gambia 2[40]	Mboto	2009	CS	urban	both	inpatient	56	-	7	-	-
Ghana 1[41]	Brandful	1999	CS	both	adult	outpatient	182	-	15	-	-
Ghana 2[42]	Kubio	2012	CS	rural	adult	blood donors	33	4	-	-	-
Ghana 3[43]	Sagoe	2012	CS	urban	adult	outpatient	138	-	5	-	-
Ghana 4[44]	Saito	1999	CS	urban	both	outpatient	85	-	-	-	1
Ghana 5[45]	King	2015	CS	urban	adult	outpatient	408	-	-	-	4
Guinea-Bissau 1[46]	Plamondon	2007	CS	urban	adult	community	48	-	0	0	0
Guinea-Bissau 2[47]	Honge	2014	CS	urban	adult	outpatient	472	-	17	-	6
Ivory Coast 1[48]	Combe	2001	CS	urban	adult	outpatient	200	-	5	-	-
Ivory Coast 2[49]	Rouet	2004	RCT	urban	adult	pregnant	501	-	27	6	-
Ivory Coast 3[50]	Rouet	2008	CO	urban	paediatric	outpatient	280	-	0	-	-
Kenya 1[51]	Chakraborty	2003	CS	urban	paediatric	inpatient	60	-	-	-	0

Study	First author	Year	Design	Setting	Age Category	Population	HIV-infected	Rapid test Positive	Serology Positive	Immunoblot Positive	PCR Positive
Kenya 2[52]	Harania	2008	CO	urban	both	outpatient	378	-	5	-	-
Kenya 3[53]	Kauru	2005	CS	urban	both	inpatient	458	-	17	-	-
Kenya 4a[54]	Kauru	2005	CS	urban	both	blood donors	66	-	1	-	-
Kenya 4b[54]	Kauru	2005	CS	urban	adult	outpatient	33	-	0	-	-
Kenya 5[55]	Kerubo	2015	CS	urban	adult	community	268	6	-	-	-
Kenya 6[56]	Muriuki	2013	CS	urban	both	outpatient	300	-	34	-	-
Kenya 7[57]	Mwatelah	2015	CS	urban	adult	special	133	-	-	-	24
Lesotho 1[58]	Rabeneau	2010	CS	rural	both	outpatient	205	-	1	-	1
Lybia 1[59]	Mirzoyan	2013	CS	urban	adult	special	294	-	283	-	-
Lybia 2[60]	Ziglam	2012	CS	urban	adult	special	1128	-	948	-	-
Malawi 1[61]	Ahmed	1998	CS	rural	unknown	pregnant	50	-	6	-	-
Malawi 2[62]	Chasela	2012	RCT	urban	both	pregnant	2041	-	110	2	0
Malawi 3[63]	Moore	2010	CO	urban	adult	outpatient	300	-	-	22	-
Malawi 4[64]	Nyirenda	2008	CS	urban	both	inpatient	172	-	-	10	-
Malawi 5[65]	Sutcliffe	2002	CC	urban	adult	outpatient	279	-	35	-	-
Malawi 6[66]	Andreotti	2014	CO	unknown	adult	pregnant	309	-	8	-	1
Mali 1[67]	Bouare	2012	CO	urban	adult	pregnant	41	-	-	0	-
Mali 2[68]	Kone	2012	CS	unknown	adult	blood donors	26	0	-	-	-
Morocco 1[69]	Benjelloun	1996	CS	unknown	adult	community	116	-	-	23	-
Morocco 2[70]	Rebbani	2013	CS	urban	adult	outpatient	503	-	27	-	19
Mozambique 1[71]	Cunha	2007	CS	urban	adult	blood donors	217	-	3	-	-
Mozambique 2[72]	Naniche	2011	CO	rural	adult	outpatient	132	-	8	-	-
Mozambique 3[73]	Rodrigues	2008	CS	urban	adult	blood donors	300	-	-	-	47
Mozambique 4[74]	Stokx	2011	CS	rural	adult	blood donors	58	6	-	0	-
Namibia 1[75]	Mavenyengwa	2014	CS	urban	adult	blood donors	75	-	-	-	1
Niger 1[76]	Mayaki	2013	CS	unknown	adult	blood donors	52	-	1	-	-
Nigeria 1[77]	Adekunle	2011	CS	urban	adult	outpatient	273	2	-	-	-
Nigeria 2[78]	Adewole	2009	CS	urban	adult	outpatient	260	-	10	-	-
Nigeria 3[79]	Adoga	2009	CS	urban	adult	special	54	-	0	-	-

Study	First author	Year	Design	Setting	Age Category	Population	HIV-infected	Rapid test Positive	Serology Positive	Immunoblot Positive	PCR Positive
Nigeria 4[80]	Agbaji	2013	CO	urban	adult	outpatient	1431	-	262	-	79
Nigeria 5[81]	Agwale	2004	CS	urban	adult	outpatient	146	-	-	-	12
Nigeria 6[82]	Akinbame	2010	CS	urban	adult	outpatient	267	9	-	-	-
Nigeria 7[83]	Akinleye	2013	CS	urban	adult	blood donors	62	0	-	-	-
Nigeria 8[84]	Anigilaje	2013	CS	urban	paediatric	outpatient	395	-	9	-	-
Nigeria 9[85]	Balogun	2012	CS	urban	adult	outpatient	102	-	19	-	-
Nigeria 10[86]	Buseri	2009	CS	urban	adult	blood donors	44	-	4	-	-
Nigeria 11[87]	Diwe	2013	CO	both	adult	outpatient	404	3	-	-	-
Nigeria 12[88]	Fasola	2008	CS	urban	adult	blood donors	2557	-	53	-	-
Nigeria 13[89]	Forbi	2007	CS	urban	adult	outpatient	180	-	33	-	-
Nigeria 14[90]	Gwanzhi	2013	CO	urban	adult	outpatient	17882	-	2467	-	-
Nigeria 15[91]	Inyama	2005	CS	urban	adult	outpatient	490	-	28	-	-
Nigeria 16[92]	Jeremiah	2009	CS	urban	adult	community	21	-	0	-	-
Nigeria 17[93]	Ladep	2007	CS	urban	both	outpatient	1044	-	90	-	-
Nigeria 18[94]	Ladep	2013	CO	urban	adult	outpatient	17882	-	2467	-	-
Nigeria 19[95]	Lesi	2007	CC	urban	adult	outpatient	120	-	7	-	-
Nigeria 20[96]	Mabayoje	2007	CS	urban	adult	blood donors	104	12	-	-	-
Nigeria 21[97]	Mabayoje	2013	CS	urban	both	outpatient	280	-	65	-	-
Nigeria 22[98]	Obienu	2011	CS	urban	adult	outpatient	180	-	12	-	-
Nigeria 23[99]	Odama	2006	CS	unknown	adult	outpatient	363	-	43	-	-
Nigeria 24[100]	Okeke	2012	CS	urban	adult	pregnant	401	-	-	-	4
Nigeria 25[101]	Okwuraiwe	2012	CS	urban	adult	outpatient	10214	-	387	-	-
Nigeria 26[102]	Olokoba	2008	CS	urban	adult	outpatient	200	9	-	-	-
Nigeria 27[103]	Omosigho	2011	CS	urban	adult	outpatient	250	19	-	-	-
Nigeria 28[104]	Onakewhor	2009	CS	urban	adult	pregnant	23	-	0	-	-
Nigeria 29[105]	Opaleye	2014	CS	urban	both	outpatient	96	-	2	-	-
Nigeria 30[106]	Otegbayo	2008	CS	urban	both	outpatient	1779	-	104	-	-
Nigeria 31[107]	Sadoh	2011	CS	urban	paediatric	outpatient	155	-	8	-	-
Nigeria 32[108]	Tremeau	2012	CS	urban	adult	outpatient	443	-	13	-	-

Study	First author	Year	Design	Setting	Age Category	Population	HIV-infected	Rapid test Positive	Serology Positive	Immunoblot Positive	PCR Positive
Nigeria 33[109]	Ya'Aba	2009	CS	urban	adult	pregnant	203	-	-	5	-
Nigeria 34[110]	Agaba	2014	CO	urban	adult	outpatient	14487	-	1934	-	-
Nigeria 35[111]	Ezechi	2014	CS	urban	both	pregnant	2391	-	37	-	-
Nigeria 36[112]	Newton	2015	CS	both	both	outpatient	200	30	-	-	-
Nigeria 37[113]	Peter	2015	CS	urban	adult	outpatient	232	-	9	-	7
Rwanda 1[114]	Pirillo	2007	RCT	urban	adult	pregnant	82	-	4	-	-
Rwanda 2[115]	Rusine	2013	CO	urban	adult	outpatient	402	-	23	-	-
Senegal 1[116]	Diop-Ndiaye	2008	CO	urban	adult	outpatient	362	-	29	6	-
Senegal 2[117]	Leprêtre	2015	CS	urban	adult	special	22	-	13	-	-
South Africa 1[118]	Barth	2011	CS	rural	adult	outpatient	248	-	-	2	-
South Africa 2[119]	Duplessis	1999	CS	urban	both	special	30	-	-	1	-
South Africa 3[120]	Gededzha	2010	CS	urban	adult	outpatient	653	-	8	-	1
South Africa 4[121]	Hoffmann	2007	CO	urban	adult	outpatient	53	-	0	-	-
South Africa 5[122]	Hoffmann	2012	CO	urban	adult	outpatient	981	-	1	-	-
South Africa 6[123]	Lodenyo	2000	CS	urban	adult	inpatient	100	-	1	-	-
South Africa 7[124]	Parboosing	2008	CS	unknown	both	inpatient	778	-	104	-	-
South Africa 8[125]	Soni	1993	CS	unknown	adult	special	20	-	0	-	-
Sudan[126]	Mudawi	2014	CS	urban	adult	outpatient	358	-	6	-	-
Tanzania 1[127]	Bowring	2013	CS	urban	adult	special	159	47	-	-	-
Tanzania 2[128]	Franzeck	2013	CO	rural	adult	outpatient	272	-	10	-	-
Tanzania 3[129]	Johnston	2010	CS	urban	both	special	65	-	49	-	-
Tanzania 4[130]	Kitundu	2001	CS	urban	paediatric	outpatient	25	-	0	-	-
Tanzania 5[131]	Matee	2006	CS	urban	adult	blood donors	58	-	0	-	-
Tanzania 6[132]	Menendez	1999	CS	rural	adult	pregnant	66	-	-	-	1
Tanzania 7[133]	Msuya	2006	CS	urban	adult	pregnant	44	-	-	1	-
Tanzania 8[134]	Mugusi	2012	CO	urban	adult	outpatient	473	-	12	-	-
Tanzania 9[135]	Nagu	2008	CS	urban	adult	outpatient	260	-	47	-	-
Tanzania 10[136]	Telatela	2007	CS	urban	paediatric	outpatient	167	-	23	-	-
Tanzania 11[137]	Tess	2000	CS	both	both	community	22	-	-	1	-

Study	First author	Year	Design	Setting	Age Category	Population	HIV-infected	Rapid test Positive	Serology Positive	Immunoblot Positive	PCR Positive
Tanzania 12[138]	Waddell	2006	CS	urban	adult	blood donors	100	-	-	-	0
Tanzania 13[139]	Muro	2013	CO	unknown	paediatric	outpatient	157	-	0	-	-
Tchad 1[140]	Bessimbaye	2014	CS	urban	adult	outpatient	725	-	7	-	-
Togo 1a[141]	Agbodjan	1995	CS	urban	both	inpatient	58	-	-	4	3
Togo 1b[141]	Agbodjan	1995	CS	urban	both	blood donors	20	-	-	1	1
Tunisia 1[142]	Kilani	2007	CS	urban	adult	outpatient	362	-	144	-	-
Tunisia 2[143]	Maaref	2011	CS	urban	adult	outpatient	125	-	33	-	-
Uganda 1[144]	Jackson	1991	CS	urban	unknown	pregnant	96	-	0	-	-
Uganda 2[145]	Mullis	2013	CO	rural	adult	outpatient	500	-	31	-	0
Uganda 3[146]	O'Reilly	2011	CS	urban	adult	inpatient	250	6	-	-	2
Uganda 4[114]	Pirillo	2007	RCT	urban	adult	pregnant	165	-	1	-	
Uganda 5[147]	Seremba	2010	CS	urban	both	outpatient	194	10	16	-	5
Uganda 6[148]	Walusansa	2009	CS	urban	adult	outpatient	122	4	-	-	-
Uganda 7[149]	Baseke	2015	CS	urban	both	outpatient	89	-	6	-	-
Zambia 1[150]	Kapembwa	2011	CS	urban	adult	outpatient	323	-	4	-	-
Zambia 2[151]	Oshitani	1995	CS	urban	adult	inpatient	182	-	1	-	1
Zimbabwe[152]	Kallestrup	2003	CO	rural	adult	outpatient	124	-	-	1	-

CS: cross-sectional, CO: cohort, RCT: randomized controlled trial, CC: case-control

Supplementary table 2: studies in special populations

Population	Country	Author	Year	HIV-infected	HIV/HCV Prevalence in % (test)
**Prisoners**	Libya	Ziglam	2012	1128	84.0 (serology)
	Nigeria	Adoga	2009	54	0 (serology)
**PWID**	Libya	Mirzoyan	2013	294	96.3 (serology)
	Kenya	Mwatelah	2015	133	18.0 (PCR)
	Senegal	Leprêtre	2015	22	59.1 (serology)
	Tanzania	Bowring	2013	159	29.6 (rapid test)
**MSM (+- PWID)**	Zanzibar	Johnston	2010	65	75.4 (serology)
**MSM**	RSA	Soni	1993	20	0 (serology)
**Female sex workers**	Ethiopia	Ayele	2002	150	5.3 (immunoblot)
	DR Congo	Laurent	2001	390	7.4 (immunoblot)
**Forensic cases**	RSA	Duplessis	1999	30	3.3 (immunoblot)

PWID: persons who inject drugs; MSM: men who have sex with men; RSA: Republic of South Africa

Supplementary table 3: Studies with available data on HIV/HCV prevalence from serology and PCR

Country	Author	Number of HIV-infected	HIV/HCV prevalence		N. of anti-HCV+ with PCR values available	Correction factor**
			Serology	PCR		
**North Africa**			**5.4**	**3.8**		**1.42**
Morocco	Rebbani	503	5.4	3.8	27/27	
**West Africa**			**12.3**	**2.1**		**3.69**
Burkina Faso	Simpore 05	58	15.5	0	7/9***	
Gambia	Jobarteh	572	11.0	0.7	7/63***	
Guinea-Bissau	Honge	472	3.6	1.3	17/17	
Ivory Coast	Rouet 04	501	5.4	1.6	10/27***	
Nigeria	Agbaji	1,431	18.3	5.5	262/262	
Nigeria	Peter	232	3.9	3.0	9/9	
**Central Africa**			**16.9**	**11.6**		**1.43**
Cameroon	Laurent 10	169	16.6	12.4	28/28	
Cameroon	Nkengasong	80	17.5	10.0	12/14	
Gabon	Rouet	762	8.8	6.2	67/67	
**East Africa**			**6.7**	**1.0**		**5.71**
Ethiopia	Abreha	734	6.8	1.5	50/50	
Uganda	Mullis	500	6.2	0	31/31	
Uganda	Seremba	194	8.2	2.6	26/26	
**Southern Africa**			**2.1**	**0**		**25.60**
Lesotho	Rabeneau	205	0.5	0.5	1/1	
Malawi	Chasela	2,041	5.4	0	109/110	
Malawi	Andreotti	309	2.6	0.3	8/8	
South Africa	Gededzha	653	1.2	0.2	8/8	
Zambia	Oshitani	182	0.5	0.5	1/1	

*From meta-regression, by region

** Correction factor for prevalence of replicating HCV infection

***all patients with positive immunoblot were tested with PCR.

Supplementary table 4: Effect of study characteristics on anti-HCV antibody prevalence, by random-effect meta-regression

		Univariable analysis		Multivariable analysis	
Study characteristic	Total (%)	Coefficient (95% CI)	P-value	Coefficient (95% CI)	P-value
Study design			0.35		
RCT	4 (3.1)	Ref.			
Cohort study	26 (19.8)	0.02 (-0.13-0.17)			
Cross-sectional	101 (77.1)	0.06 (-0.09-0.20)			
Year of publication			0.29		
Before 2000	6 (4.7)	Ref.			
2000-2009	45 (34.8)	0.01 (-0.12-0.13)			
After 2009	78 (60.5)	0.04 (-0.08-0.16)			
Study population			<0.001		<0.001
Outpatients	75 (58.1)	Ref.		Ref.	
Inpatients	14 (10.9)	-0.03 (-0.09-0.02)		-0.02 (-0.07-0.04)	
Blood donors	19 (14.7)	-0.01 (-0.07-0.04)		-0.001 (-0.05-0.05)	
Pregnant women	9 (7.0)	-0.03 (-0.11-0.04)		-0.01 (-0.08-0.07)	
Community	4 (3.1)	-0.05 (-0.16-0.05)		-0.04 (-0.14-0.05)	
Special populations	8 (6.2)	0.41 (0.32-0.50)		0.35 (0.26-0.43)	
Age category			0.56		
Adults	97 (76.4)	Ref.			
Children	8 (6.3)	-0.05 (-0.16-0.05)			
Both	22 (17.3)	0.01 (-0.06-0.07)			
Setting			0.79		
Urban	100 (84.7)	Ref.			
Rural	7 (5.9)	-0.02 (-0.14-0.09)			
Both	11 (9.4)	-0.03 (-0.12-0.06)			
Region			<0.001		<0.001
Central	12 (9.3)	Ref.			Ref.
East	34 (26.3)	0.01 (-0.08-0.08)		-0.02 (-0.08-0.05)	
North	6 (4.7)	0.35 (0.23-0.47)		0.23 (0.13-0.33)	
Southern	19 (14.7)	-0.03 (-0.12-0.05)		-0.05 (-0.12-0.03)	
West	58 (45.0)	-0.01 (0.00-0.10)		-0.01 (-0.07-0.05)	

Supplementary table 5: HIV/HCV-coinfection prevalence from serology and PCR including imputed PCR prevalence estimates using the correction factor (after exclusion of studies in special populations)

Country	HIV/HCV-coinfection prevalence		
	Serology	PCR (original)	PCR (imputed)
**North Africa**			
Morocco	5.4 (3.3-7.4)	3.8 (2.0-5.6)	
Tunisia	33.5 (20.4-46.6)		23.6
Sudan	1.7 (0.2-3.2)		1.2
**West Africa**			
Burkina Faso	7.4 (4.3-10.5)	1.6 (0-3.8)	
Gambia	11.1 (8.6-13.7)	0.7 (0-1.5)	
Ghana	6.4 (2.2-10.5)	1.0 (0-2.1)	
Guinea-Bissau	2.2 (0-5.6)	1.3 (0.1-2.4)	
Ivory Coast	2.5 (0-6.1)	1.6 (0.4-2.8)	
Mali	0 (0-6.7)		0
Niger	1.9 (0-7.0)		0.5
Nigeria	7.2 (5.1-9.3)	4.0 (1.0-7.0)	
Senegal	8.0 (5.1-10.9)		2.2
Togo		5.1 (0-11.0)	
**Central Africa**			
Cameroon	8.1 (4.0-12.2)	9.4 (5.6-13.2)	
Tchad	1.0 (0.2-1.8)		0.7
DRC	9.6 (5.3-13.8)		6.7
Equatorial Guinea	3.5 (0.9-6.1)		2.4
Gabon	8.8 (6.7-10.9)	6.2 (4.4-7.9)	
Rwanda	5.6 (3.4-7.8)		3.9
**East Africa**			
Ethiopia	6.2 (3.8-8.6)	1.5 (0.5-2.5)	
Kenya	3.3 (1.0-5.7)	0 (0-2.5)	
Tanzania	5.0 (1.6-8.4)	0.3 (0-1.9)	
Uganda	3.1 (1.0-5.1)	0.6 (0-1.8)	
**Southern Africa**			
Angola	23.7 (9.3-38.1)		0.9

Botswana	0.7 (0-2.0)		0.03
Lesotho	0.5 (0-1.8)	0.5 (0-1.8)	
Malawi	6.8 (3.3-10.4)	0 (0-0.1)	
Mozambique	4.7 (0-9.5)	15.7 (11.4-19.9)	
Namibia		1.3 (0-4.9)	
South Africa	3.1 (0.1-6.1)	0.2 (0-0.6)	
Zambia	0.9 (0-1.9)	0.5 (0-2.1)	

Supplementary figure 1. Meta-analysis of Hepatitis C prevalence among HIV-infected individuals from outpatient clinics, by country, according to anti-HCV antibody tests (A) and PCR (B) results

A

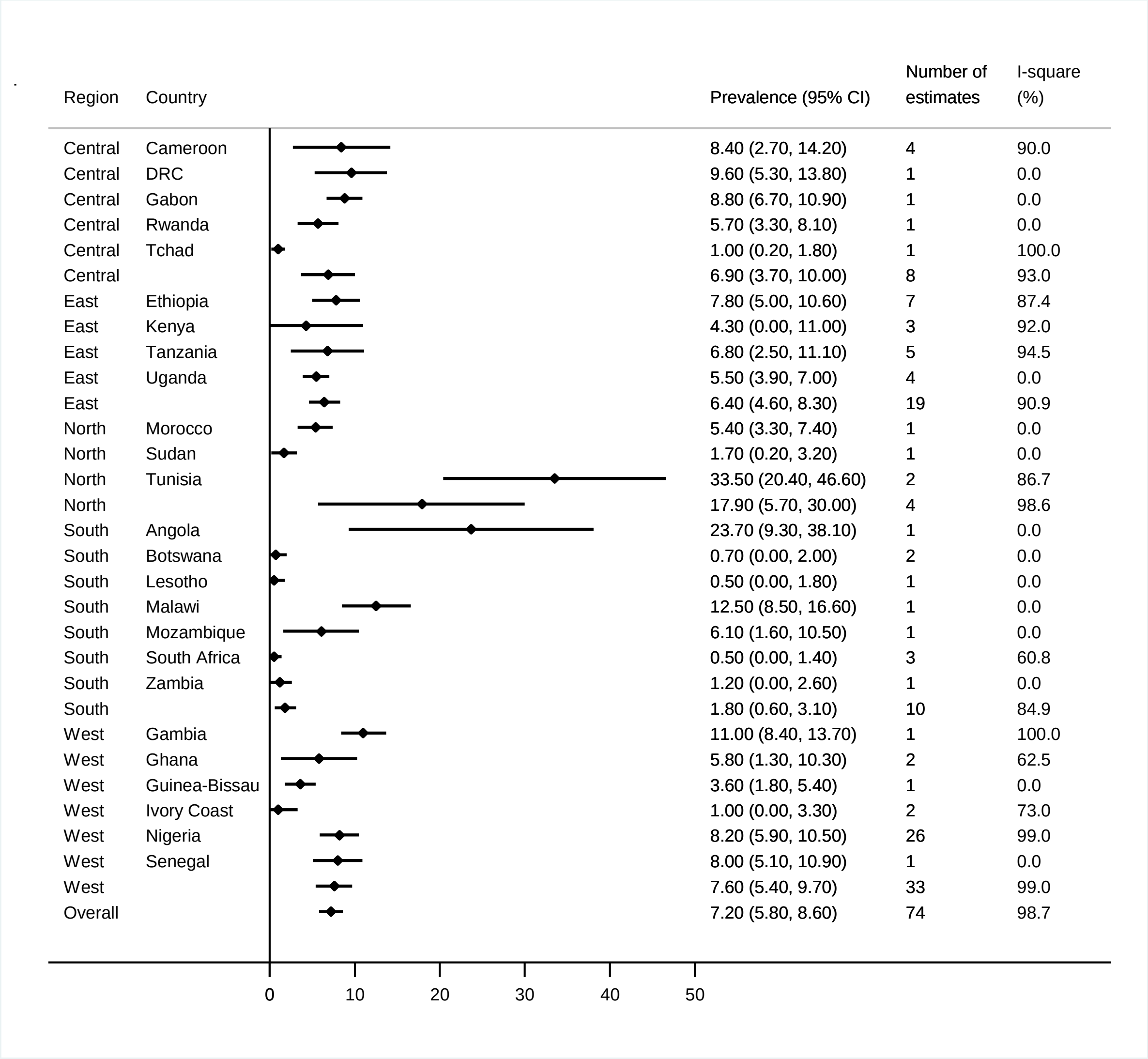

B

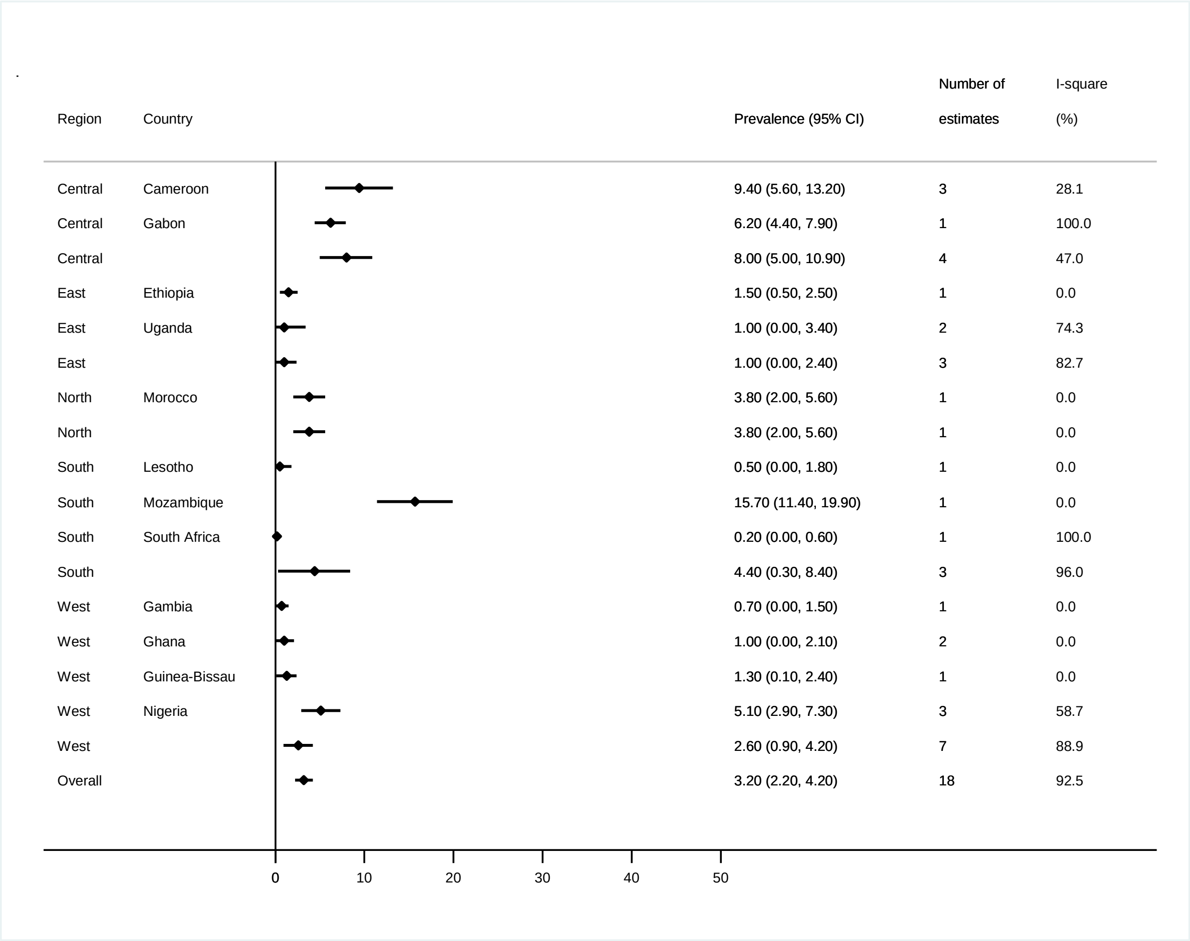
Region Country Prevalence (95% CI) Number of estimates I-square (%)
Central Cameroon 9.40 (5.60, 13.20) 3 28.1
Central Gabon 6.20 (4.40, 7.90) 1 100.0
Central 8.00 (5.00, 10.90) 4 47.0
East Ethiopia 1.50 (0.50, 2.50) 1 0.0
East Uganda 1.00 (0.00, 3.40) 2 74.3
East 1.00 (0.00, 2.40) 3 82.7
North Morocco 3.80 (2.00, 5.60) 1 0.0
North 3.80 (2.00, 5.60) 1 0.0
South Lesotho 0.50 (0.00, 1.80) 1 0.0
South Mozambique 15.70 (11.40, 19.90) 1 0.0
South South Africa 0.20 (0.00, 0.60) 1 100.0
South 4.40 (0.30, 8.40) 3 96.0
West Gambia 0.70 (0.00, 1.50) 1 0.0
West Ghana 1.00 (0.00, 2.10) 2 0.0
West Guinea-Bissau 1.30 (0.10, 2.40) 1 0.0
West Nigeria 5.10 (2.90, 7.30) 3 58.7
West 2.60 (0.90, 4.20) 7 88.9
Overall 3.20 (2.20, 4.20) 18 92.5
0
10
20
30
40
50

References

1. Guimaraes Nebenzahl H, Lopes A, Castro R, Pereira F. Prevalence of human immunodeficiency virus, hepatitis C virus, hepatitis B virus and syphilis among individuals attending anonymous testing for HIV in Luanda, Angola. *South African medical journal = Suid-Afrikaanse tydskrif vir geneeskunde* 2013; **103**(3): 186-8.
2. Patel P, Davis S, Tolle M, Mabikwa V, Anabwani G. Prevalence of hepatitis B and hepatitis C coinfections in an adult HIV centre population in Gaborone, Botswana. *The American journal of tropical medicine and hygiene* 2011; **85**(2): 390-4.
3. Wester CW, Bussmann H, Moyo S, et al. Serological evidence of HIV-associated infection among HIV-1-infected adults in Botswana. *Clinical infectious diseases : an official publication of the Infectious Diseases Society of America* 2006; **43**(12): 1612-5.
4. Collenberg E, Ouedraogo T, Ganame J, et al. Seroprevalence of six different viruses among pregnant women and blood donors in rural and urban Burkina Faso: A comparative analysis. *Journal of medical virology* 2006; **78**(5): 683-92.
5. Kania D, Sangare L, Sakande J, et al. A new strategy to improve the cost-effectiveness of human immunodeficiency virus, hepatitis B virus, hepatitis C virus, and syphilis testing of blood donations in sub-Saharan Africa: a pilot study in Burkina Faso. *Transfusion* 2009; **49**(10): 2237-40.
6. Nagalo MB, Sanou M, Bisseye C, et al. Seroprevalence of human immunodeficiency virus, hepatitis B and C viruses and syphilis among blood donors in Koudougou (Burkina Faso) in 2009. *Blood transfusion = Trasfusione del sangue* 2011; **9**(4): 419-24.
7. Nagalo BM, Bisseye C, Sanou M, et al. Seroprevalence and incidence of transfusion-transmitted infectious diseases among blood donors from regional blood transfusion centres in Burkina Faso, West Africa. *Tropical medicine & international health : TM & IH* 2012; **17**(2): 247-53.
8. Simpore J, Granato M, Santarelli R, et al. Prevalence of infection by HHV-8, HIV, HCV and HBV among pregnant women in Burkina Faso. *Journal of clinical virology : the official publication of the Pan American Society for Clinical Virology* 2004; **31**(1): 78-80.
9. Simpore J, Ilboudo D, Samandoulougou A, Guardo P, Castronovo P, Musumeci S. HCV and HIV co-infection in pregnant women attending St. Camille Medical Centre in Ouagadougou (Burkina Faso). *Journal of medical virology* 2005; **75**(2): 209-12.
10. Simpore J, Savadogo A, Ilboudo D, et al. Toxoplasma gondii, HCV, and HBV seroprevalence and co-infection among HIV-positive and -negative pregnant women in Burkina Faso. *Journal of medical virology* 2006; **78**(6): 730-3.
11. Zeba MT, Karou SD, Sagna T, et al. HCV prevalence and co-infection with HIV among pregnant women in Saint Camille Medical Centre, Ouagadougou. *Tropical medicine & international health : TM & IH* 2011; **16**(11): 1392-6.
12. Kirakoya-Samadoulougou F, Sanou M, Samadoulougou S, et al. High seroprevalence of hepatitis B virus and hepatitis C virus among human immunodeficiency virus carriers in blood donors of Burkina Faso: A need for their screening before HARRT therapy. *Journal of viral hepatitis* 2014; **21**(7): e52-e63.
13. Ntakarutimana V, Delaporte E, Pollet D, Demedts P, Scharpe S. [Seroprevalence of hepatitis C virus among persons visiting the Burundi health services]. *Annales de la Societe belge de medecine tropicale* 1995; **75**(4): 283-90.
14. Feldt T, Sarfo FS, Zoufaly A, et al. Hepatitis E virus infections in HIV-infected patients in Ghana and Cameroon. *Journal of clinical virology : the official publication of the Pan American Society for Clinical Virology* 2013; **58**(1): 18-23.

15. Laurent C, Bourgeois A, Mpoudi M, Butel C, Mpoudi-Ngole E, Delaporte E. HIV and hepatitis C virus coinfection, Cameroon. *Emerging infectious diseases* 2007; **13**(3): 514-6.
16. Laurent C, Bourgeois A, Mpoudi-Ngole E, et al. High rates of active hepatitis B and C co-infections in HIV-1 infected Cameroonian adults initiating antiretroviral therapy. *HIV medicine* 2010; **11**(1): 85-9.
17. Mbanya DN, Takam D, Ndumbe PM. Serological findings amongst first-time blood donors in Yaounde, Cameroon: is safe donation a reality or a myth? *Transfusion medicine (Oxford, England)* 2003; **13**(5): 267-73.
18. Ndjomou J, Kupfer B, Kochan B, Zekeng L, Kaptue L, Matz B. Hepatitis C virus infection and genotypes among human immunodeficiency virus high-risk groups in Cameroon. *Journal of medical virology* 2002; **66**(2): 179-86.
19. Nkengasong JN, Claeys H, De Beenhouwer H, et al. Hepatitis C virus antibody, viraemia and genotypes in individuals infected with HIV-1 in Cameroon. *Annales de la Societe belge de medecine tropicale* 1994; **74**(3): 249-52.
20. Noubiap JJ, Joko WY, Nansseu JR, Tene UG, Siaka C. Sero-epidemiology of human immunodeficiency virus, hepatitis B and C viruses, and syphilis infections among first-time blood donors in Edea, Cameroon. *International journal of infectious diseases : IJID : official publication of the International Society for Infectious Diseases* 2013.
21. Noubiap JJ, Aka PV, Nanfack AJ, Agyingi LA, Ngai JN, Nyambi PN. Hepatitis B and C Co-Infections in Some HIV-Positive Populations in Cameroon, West Central Africa: Analysis of Samples Collected Over More Than a Decade. *PloS one* 2015; **10**(9): e0137375.
22. Pawlotsky JM, Belec L, Gresenguet G, et al. High prevalence of hepatitis B, C, and E markers in young sexually active adults from the Central African Republic. *Journal of medical virology* 1995; **46**(3): 269-72.
23. Kabinda JM, Katchunga BP. Viral hepatitis B and C in individuals infected with human immunodeficiency virus in Bukavu (South-Kivu), Democratic Republic of Congo. [French]

Les hepatites virales B et C chez les porteurs du virus de l'immunodeficience humaine a Bukavu (Sud-Kivu), Republique democratique du Congo. *Journal Africain d'Hepato-Gastroenterologie* 2010; **4**(4): 230-5.
24. Laurent C, Henzel D, Mulanga-Kabeya C, Maertens G, Larouze B, Delaporte E. Seroepidemiological survey of hepatitis C virus among commercial sex workers and pregnant women in Kinshasa, Democratic Republic of Congo. *International journal of epidemiology* 2001; **30**(4): 872-7.
25. Xie DD, Li J, Chen JT, et al. Seroprevalence of Human Immunodeficiency Virus, Hepatitis B Virus, Hepatitis C Virus, and Treponema pallidum Infections among Blood Donors on Bioko Island, Equatorial Guinea. *PloS one* 2015; **10**(10): e0139947.
26. Abreha T, Woldeamanuel Y, Pietsch C, et al. Genotypes and viral load of hepatitis C virus among persons attending a voluntary counseling and testing center in Ethiopia. *Journal of medical virology* 2011; **83**(5): 776-82.
27. Ayele W, Nokes DJ, Abebe A, et al. Higher prevalence of anti-HCV antibodies among HIV-positive compared to HIV-negative inhabitants of Addis Ababa, Ethiopia. *Journal of medical virology* 2002; **68**(1): 12-7.
28. Diro E, Alemu S, A GY. Blood safety & prevalence of transfussion transmissible viral infections among donors at the Red Cross Blood Bank in Gondar University Hospital. *Ethiopian medical journal* 2008; **46**(1): 7-13.
29. Tessema B, Yismaw G, Kassu A, et al. Seroprevalence of HIV, HBV, HCV and syphilis infections among blood donors at Gondar University Teaching Hospital, Northwest Ethiopia: declining trends over a period of five years. *BMC infectious diseases* 2010; **10**: 111.
30. Tiruneh M. Seroprevalence of multiple sexually transmitted infections among antenatal clinic attendees in Gondar Health Center, northwest Ethiopia. *Ethiopian medical journal* 2008; **46**(4): 359-66.

31. Balew M, Moges F, Yismaw G, Unakal C. Assessment of hepatitis B virus and hepatitis C virus infections and associated risk factors in HIV infected patients at Debretabor hospital, South Gondar, Northwest Ethiopia. *Asian Pacific Journal of Tropical Disease* 2014; **4**(1): 1-7.
32. Hadush H, Gebre-Selassie S, Mihret A. Hepatitis C virus and human immunodeficiency virus coinfection among attendants of voluntary counseling and testing centre and HIV follow up clinics in Mekelle Hospital. *The Pan African medical journal* 2013; **14**: 107.
33. Manyazewal T, Sisay Z, Biadgilign S, Abegaz WE. Hepatitis B and hepatitis C virus infections among antiretroviral-naive and -experienced HIV co-infected adults. *Journal of medical microbiology* 2014; **63**(Pt 5): 742-7.
34. Taye S, Lakew M. Impact of hepatitis C virus co-infection on HIV patients before and after highly active antiretroviral therapy: an immunological and clinical chemistry observation, Addis Ababa, Ethiopia. *BMC immunology* 2013; **14**: 23.
35. Wondimeneh Y, Alem M, Asfaw F, Belyhun Y. HBV and HCV seroprevalence and their correlation with CD4 cells and liver enzymes among HIV positive individuals at University of Gondar Teaching Hospital, Northwest Ethiopia. *Virology journal* 2013; **10**: 171.
36. Abera B, Zenebe Y, Mulu W, Kibret M, Kahsu G. Seroprevalence of hepatitis B and C viruses and risk factors in HIV infected children at the Felgehiwot referral hospital, Ethiopia. *BMC Res Notes* 2014; **7**: 838.
37. Mulu W, Gidey B, Chernet A, Alem G, Abera B. Hepatotoxicity and associated risk factors in HIV-infected patients receiving antiretroviral therapy at Felege Hiwot Referral Hospital, Bahirdar, Ethiopia. *Ethiop J Health Sci* 2013; **23**(3): 217-26.
38. Rouet F, Deleplancque L, Mboumba BB, et al. Usefulness of a fourth generation ELISA assay for the reliable identification of HCV infection in HIV-positive adults from Gabon (Central Africa). *PloS one* 2015; **10**(1): e0116975.
39. Jobarteh M, Malfroy M, Peterson I, et al. Seroprevalence of hepatitis B and C virus in HIV-1 and HIV-2 infected Gambians. *Virology journal* 2010; **7**: 230.
40. Mboto CI, Davies-Russell A, Fielder M, Jewell AP. CD4+ lymphocyte values and trends in individuals infected with human immunodeficiency virus and/or co-infected with hepatitis C virus in the Gambia. *African health sciences* 2009; **9**(3): 130-6.
41. Brandful JA, Apeagyei FA, Ampofo WK, et al. Relationship between immunoclinical status and prevalence of viral sexually transmitted diseases among human immunodeficiency virus-1 seropositive patients in Ghana. *Viral immunology* 1999; **12**(2): 131-7.
42. Kubio C, Tierney G, Quaye T, et al. Blood transfusion practice in a rural hospital in Northern Ghana, Damongo, West Gonja District. *Transfusion* 2012; **52**(10): 2161-6.
43. Sagoe KW, Agyei AA, Ziga F, et al. Prevalence and impact of hepatitis B and C virus co-infections in antiretroviral treatment naive patients with HIV infection at a major treatment center in Ghana. *Journal of medical virology* 2012; **84**(1): 6-10.
44. Saito T, Ishikawa KI, Osei-Kwasi M, et al. Prevalence of hepatitis G virus and characterization of viral genome in Ghana. *Hepatology Research* 1999; **13**(3): 221-31.
45. King S, Adjei-Asante K, Appiah L, et al. Antibody screening tests variably overestimate the prevalence of hepatitis C virus infection among HIV-infected adults in Ghana. *Journal of viral hepatitis* 2015; **22**(5): 461-8.
46. Plamondon M, Labbe AC, Frost E, et al. Hepatitis C virus infection in Guinea-Bissau: a sexually transmitted genotype 2 with parenteral amplification? *PloS one* 2007; **2**(4): e372.
47. Honge BL, Jespersen S, Medina C, et al. Hepatitis C prevalence among HIV-infected patients in Guinea-Bissau: a descriptive cross-sectional study. *International journal of infectious diseases : IJID : official publication of the International Society for Infectious Diseases* 2014; **28**: 35-40.
48. Combe P, La Ruche G, Bonard D, et al. Hepatitis B and C infections, human immunodeficiency virus and other sexually transmitted infections among women of childbearing age in Cote d'Ivoire, West Africa. *Transactions of the Royal Society of Tropical Medicine and Hygiene* 2001; **95**(5): 493-6.
49. Rouet F, Chaix ML, Inwoley A, et al. HBV and HCV prevalence and viraemia in HIV-positive and HIV-negative pregnant women in Abidjan, Cote d'Ivoire: The ANRS 1236 study. *Journal of medical virology* 2004; **74**(1): 34-40.

50. Rouet F, Chaix ML, Inwoley A, et al. Frequent occurrence of chronic hepatitis B virus infection among West African HIV type-1 - Infected children. *Clinical Infectious Diseases* 2008; **46**(3): 361-6.
51. Chakraborty R, Rees G, Bourboulia D, et al. Viral coinfections among African children infected with human immunodeficiency virus type 1. *Clinical Infectious Diseases* 2003; **36**(7): 922-4.
52. Harania RS, Karuru J, Nelson M, Stebbing J. HIV, hepatitis B and hepatitis C coinfection in Kenya. *AIDS (London, England)* 2008; **22**(10): 1221-2.
53. Karuru JW, Lule GN, Joshi M, Anzala O. Prevalence of HCV and HIV/HCV co-infection among volunteer blood donors and VCT clients. *East African medical journal* 2005; **82**(4): 166-9.
54. Karuru JW, Lule GN, Joshi M, Anzala O. Prevalence of HCV and HCV/HIV co-infection among in-patients at the Kenyatta National Hospital. *East African medical journal* 2005; **82**(4): 170-2.
55. Kerubo G, Khamadi S, Okoth V, et al. Hepatitis B, hepatitis C and HIV-1 coinfection in two informal urban settlements in Nairobi, Kenya. *PloS one* 2015; **10 (6) (no pagination)**(e0129247).
56. Muriuki BM, Gicheru MM, Wachira D, Nyamache AK, Khamadi SA. Prevalence of hepatitis B and C viral co-infections among HIV-1 infected individuals in Nairobi, Kenya. *BMC Res Notes* 2013; **6**: 363.
57. Mwatelah RS, Lwembe RM, Osman S, et al. Co-infection burden of hepatitis C virus and human immunodeficiency virus among injecting heroin users at the kenyan coast. *PloS one* 2015; **10 (7) (no pagination)**(e0132287).
58. Rabenau HF, Lennemann T, Kircher C, et al. Prevalence- and gender-specific immune response to opportunistic infections in HIV-infected patients in Lesotho. *Sexually transmitted diseases* 2010; **37**(7): 454-9.
59. Mirzoyan L, Berendes S, Jeffery C, et al. New evidence on the HIV epidemic in Libya: why countries must implement prevention programs among people who inject drugs. *Journal of acquired immune deficiency syndromes (1999)* 2013; **62**(5): 577-83.
60. Ziglam H, Zorgani AA, Balouz A, Abudhe AH, Elahmer O. Prevalence of antibodies to human immunodeficiency virus, hepatitis B, and hepatitis C in prisoners in Libya. *Libyan Journal of Medicine* 2012; **7**(1).
61. Ahmed SD, Cuevas LE, Brabin BJ, et al. Seroprevalence of hepatitis B and C and HIV in Malawian pregnant women. *The Journal of infection* 1998; **37**(3): 248-51.
62. Chasela CS, Wall P, Drobeniuc J, et al. Prevalence of hepatitis C virus infection among human immunodeficiency virus-1-infected pregnant women in Malawi: The BAN study. *Journal of Clinical Virology* 2012; **54**(4): 318-20.
63. Moore E, Beadsworth MB, Chaponda M, et al. Favourable one-year ART outcomes in adult Malawians with hepatitis B and C co-infection. *The Journal of infection* 2010; **61**(2): 155-63.
64. Nyirenda M, Beadsworth MB, Stephany P, et al. Prevalence of infection with hepatitis B and C virus and coinfection with HIV in medical inpatients in Malawi. *The Journal of infection* 2008; **57**(1): 72-7.
65. Sutcliffe S, Taha TE, Kumwenda NI, Taylor E, Liomba GN. HIV-1 prevalence and herpes simplex virus 2, hepatitis C virus, and hepatitis B virus infections among male workers at a sugar estate in Malawi. *Journal of acquired immune deficiency syndromes (1999)* 2002; **31**(1): 90-7.
66. Andreotti M, Pirillo MF, Liotta G, et al. The impact of HBV or HCV infection in a cohort of HIV-infected pregnant women receiving a nevirapine-based antiretroviral regimen in Malawi. *BMC infectious diseases* 2014; **14**: 180.
67. Bouare N, Vaira D, Gothot A, et al. Prevalence of HIV and HCV infections in two populations of Malian women and serological assays performances. *World journal of hepatology* 2012; **4**(12): 365-73.
68. Kone MC, Sidibe ET, Malle KK, et al. [Seroprevalence of human immunodeficiency virus, hepatitis B virus and hepatitis C virus among blood donors in Segou, Mali]. *Medecine et sante tropicales* 2012; **22**(1): 97-8.
69. Benjelloun S, Bahbouhi B, Sekkat S, Bennani A, Hda N, Benslimane A. Anti-HCV seroprevalence and risk factors of hepatitis C virus infection in Moroccan population groups. *Research in virology* 1996; **147**(4): 247-55.

70. Rebbani K, Ouladlahsen A, Bensghir A, et al. Co-infections with hepatitis B and C viruses in human immunodeficiency virus-infected patients in Morocco. *Clinical Microbiology and Infection* 2013; **19**(10): E454-E7.
71. Cunha L, Plouzeau C, Ingrand P, et al. Use of replacement blood donors to study the epidemiology of major blood-borne viruses in the general population of Maputo, Mozambique. *Journal of medical virology* 2007; **79**(12): 1832-40.
72. Naniche D, Letang E, Nhampossa T, David C, Menendez C, Alonso P. Alterations in T cell subsets in human immunodeficiency virus-infected adults with co-infections in southern Mozambique. *American Journal of Tropical Medicine and Hygiene* 2011; **85**(4): 776-81.
73. Rodrigues Mda C, Viotti JB, Braga RF, Lourenco LF, Antunes CM, Lambertucci JR. HIV/HCV coinfection in Infectious Disease Units in Mozambique and Brazil: a comparative study. *Revista da Sociedade Brasileira de Medicina Tropical* 2008; **41**(5): 518.
74. Stokx J, Gillet P, De Weggheleire A, et al. Seroprevalence of transfusion-transmissible infections and evaluation of the pre-donation screening performance at the Provincial Hospital of Tete, Mozambique. *BMC infectious diseases* 2011; **11**: 141.
75. Mavenyengwa RT, Mukesi M, Chipare I, Shoombe E. Prevalence of human immunodeficiency virus, syphilis, hepatitis B and C in blood donations in Namibia. *BMC public health* 2014; **14**: 424.
76. Mayaki Z, Dardenne N, Kabo R, et al. Seroprevalence of infectious markers among blood donors in Niamey (Niger). [French]

Seroprevalence des marqueurs de l'infection chez les donneurs de sang a Niamey (Niger). *Revue d'epidemiologie et de sante publique* 2013; **61**(3): 233-40.
77. Adekunle AE, Oladimeji AA, Temi AP, Adeseye AI, Akinyeye OA, Taiwo RH. Baseline CD4+ T lymphocyte cell counts, hepatitis B and C viruses seropositivity in adults with Human Immunodeficiency Virus infection at a tertiary hospital in Nigeria. *The Pan African medical journal* 2011; **9**: 6.
78. Adewole OO, Anteyi E, Ajuwon Z, et al. Hepatitis B and C virus co-infection in Nigerian patients with HIV infection. *Journal of infection in developing countries* 2009; **3**(5): 369-75.
79. Adoga MP, Banwat EB, Forbi JC, et al. Human immunonodeficiency virus, hepatitis B virus and hepatitis C virus: sero-prevalence, co-infection and risk factors among prison inmates in Nasarawa State, Nigeria. *Journal of infection in developing countries* 2009; **3**(7): 539-47.
80. Agbaji O, Thio CL, Meloni S, et al. Impact of hepatitis C virus on HIV response to antiretroviral therapy in Nigeria. *Journal of acquired immune deficiency syndromes (1999)* 2013; **62**(2): 204-7.
81. Agwale SM, Tanimoto L, Womack C, et al. Prevalence of HCV coinfection in HIV-infected individuals in Nigeria and characterization of HCV genotypes. *Journal of clinical virology : the official publication of the Pan American Society for Clinical Virology* 2004; **31 Suppl 1**: S3-6.
82. Akinbami AA, Oshinaike OO, Adeyemo TA, Adediran A, Oshikomaiya BI, Ismail KA. Seroprevalence of hepatitis C infection in HIV patients using a rapid one-step test strip kit. *Nigerian quarterly journal of hospital medicine* 2010; **20**(3): 144-6.
83. Akmleye OM, Olamyan JAT, Akintola JO, Okoye CA, Eke CF. Blood safety and prevalence of transfusion transmissible viral infections among blood donors in Lagos, Nigeria. *International Journal of Tropical Medicine* 2013; **8**(5-6): 113-8.
84. Anigilaje EA, Olutola A. Human immunodeficiency virus and hepatitis C virus co-infection among children in an antiretroviral therapy programme in Benue. *Internet Journal of Infectious Diseases* 2013; **12**(1).
85. Balogun TM, Emmanuel S, Ojerinde EF. HIV, Hepatitis B and C viruses' coinfection among patients in a Nigerian tertiary hospital. *The Pan African medical journal* 2012; **12**: 100.
86. Buseri FI, Muhibi MA, Jeremiah ZA. Sero-epidemiology of transfusion-transmissible infectious diseases among blood donors in Osogbo, south-west Nigeria. *Blood transfusion = Trasfusione del sangue* 2009; **7**(4): 293-9.
87. Kevin Diwe C, Chidiebere Okwara E, Okezie Enwere O, Emeka Azike J, Chibuzo Nwaimo N. Sero-prevalence of hepatitis B virus and hepatitis C virus among HIV patients in a suburban University Teaching Hospital in South-East Nigeria. *Pan African Medical Journal* 2013; **16**.

88. Fasola FA, Kotila TR, Akinyemi JO. Trends in transfusion-transmitted viral infections from 2001 to 2006 in Ibadan, Nigeria. *Intervirology* 2008; **51**(6): 427-31.
89. Forbi JC, Gabadi S, Alabi R, et al. The role of triple infection with hepatitis B virus, hepatitis C virus, and human immunodeficiency virus (HIV) type-1 on CD4+ lymphocyte levels in the highly HIV infected population of North-Central Nigeria. *Memorias do Instituto Oswaldo Cruz* 2007; **102**(4): 535-7.
90. Gwamzhi Ladep N, Aladi Agaba P, Agbaji O, et al. Rates and impact of hepatitis on human immunodeficiency virus infection in a large African cohort. *World Journal of Gastroenterology* 2013; **19**(10): 1602-10.
91. Inyama PU, Uneke CJ, Anyanwu GI, Njoku OM, Idoko JH, Idoko JA. Prevalence of antibodies to Hepatitis C virus among Nigerian patients with HIV infection. *Online Journal of Health and Allied Sciences* 2005; **4**(2).
92. Jeremiah ZA, Tony-Enwin EO. Seroepidemiology of transfusion transmissible viral infection among university fresh students in Port Harcourt, Nigeria. *Hepatitis monthly* 2009; **9**(4): 276-81.
93. Ladep NG, Agbaji O, Agaba P, et al. Does immunological status affect the prevalence of Hepatitis C virus infection among HIV/AIDS patients? *Nigerian journal of medicine : journal of the National Association of Resident Doctors of Nigeria* 2007; **16**(3): 231-4.
94. Ladep NG, Agaba PA, Agbaji O, et al. Rates and impact of hepatitis on human immunodeficiency virus infection in a large African cohort. *World journal of gastroenterology : WJG* 2013; **19**(10): 1602-10.
95. Lesi OA, Kehinde MO, Oguh DN, Amira CO. Hepatitis B and C virus infection in Nigerian patients with HIV/AIDS. *The Nigerian postgraduate medical journal* 2007; **14**(2): 129-33.
96. Mabayoje VO, Oparinde DP, Akanni EO, Taiwo SS, Muhibi MA, Adebayo TO. Seroprevalence of hepatitis B and C and of human immunodeficiency virus among blood donors in south-west Nigeria. *British journal of biomedical science* 2007; **64**(4): 177-9.
97. Mabayoje VO, Muhibi MA, Akindele RA, Akinleye CA, Mabayoje PS, Babatunde OS. Hepatitis C virus co-infection among people living with HIV/AIDS in a Nigerian Teaching Hospital. *HIV and AIDS Review* 2013; **12**(4): 102-5.
98. Obienu O, Nwokediuko S. Selected biochemical and hematological abnormalities in Nigerians with human immunodeficiency virus and hepatitis C virus coinfection. *Hepatic Medicine: Evidence and Research* 2011; **3**: 63-9.
99. Odama LE, Momoh-Negedu OR, Mohammed SB, et al. Prevalence of hepatitis C virus among human immunodeficiency virus infected patients and blood donors in Nigeria. *Journal of Phytomedicine and Therapeutics* 2006; **8-11**: 29-32.
100. Okeke TC, Obi SN, Okezie OA, et al. Coinfection with hepatitis B and C viruses among HIV positive pregnant women in Enugu south east, Nigeria. *Nigerian journal of medicine : journal of the National Association of Resident Doctors of Nigeria* 2012; **21**(1): 57-60.
101. Okwuraiwe AP, Audu RA, Salu OB, et al. Immunological and virological response to HAART in HIV-1 patients co-Infected with hepatitis B and C viruses

Reponse immunologique et virale au traitement antiretroviral hautement actif chez des patients VIH-1 co infectes par les virus de l' hepatite B et C. *West African journal of medicine* 2012; **31**(2): 124-8.
102. Olokoba AB, Olokoba LB, Salawu FK, et al. Hepatitis C virus and human immunodeficiency virus co-infection in North-Eastern Nigeria. *Research Journal of Medical Sciences* 2008; **2**(5): 217-9.
103. Omosigho OP, Inyinbor HE, Emumwen EG, et al. Hepatitis C virus co-infection in human immuno deficiency virus positive population in Bida, North Central Nigeria. *Internet Journal of Infectious Diseases* 2011; **9**(2).
104. Onakewhor JU, Okonofua FE. The prevalence of dual human immunodeficiency virus/hepatitis C virus (HIV/HCV) infection in asymptomatic pregnant women in Benin City, Nigeria. *African journal of reproductive health* 2009; **13**(2): 97-108.
105. Opaleye OO, Oluremi AS, Atiba AB, et al. Occult hepatitis B virus infection among HIV positive patients in nigeria. *Journal of tropical medicine* 2014; **2014**(796121).

106. Otegbayo JA, Taiwo BO, Akingbola TS, et al. Prevalence of hepatitis B and C seropositivity in a Nigerian cohort of HIV-infected patients. *Annals of hepatology* 2008; **7**(2): 152-6.
107. Sadoh AE, Sadoh WE, Iduoriyekemwen NJ. HIV co-infection with hepatitis B and C viruses among Nigerian children in an antiretroviral treatment programme. *SAJCH South African Journal of Child Health* 2011; **5**(1): 7-10.
108. Tremeau-Bravard A, Ogbukagu IC, Ticao CJ, Abubakar JJ. Seroprevalence of hepatitis B and C infection among the HIV-positive population in Abuja, Nigeria. *African health sciences* 2012; **12**(3): 312-7.
109. Ya'Aba Y, Isu NR, Mohammed SB, et al. Prevalence of Hepatitis C Virus (HCV) and Human Immunodeficiency Virus (HIV) Co-infection among pregnant women attending antenatal clinics in Abuja, Nigeria. *Journal of Phytomedicine and Therapeutics* 2009; **14**: 45-8.
110. Agaba PA, Meloni ST, Sule HM, et al. Patients who present late to HIV care and associated risk factors in Nigeria. *HIV Med* 2014; **15**(7): 396-405.
111. Ezechi OC, Kalejaiye OO, Gab-Okafor CV, et al. Sero-prevalence and factors associated with Hepatitis B and C co-infection in pregnant Nigerian women living with HIV infection. *Pan Afr Med J* 2014; **17**: 197.
112. Newton OE, Oghene OA, Okonko IO. Anti-HCV antibody among newly diagnosed HIV patients in Ughelli, a suburban area of Delta State Nigeria. *African health sciences* 2015; **15**(3): 728-36.
113. Peter YJ, Olayinka AT, Agbaji OO, Ogunsola FT. Epidemiology of hepatitis B and hepatitis C virus infections among HIV counseling and testing clients in Jos, north central Nigeria. *African Journal of Clinical and Experimental Microbiology* 2015; **16**(3): 92-6.
114. Pirillo MF, Bassani L, Germinario EA, et al. Seroprevalence of hepatitis B and C viruses among HIV-infected pregnant women in Uganda and Rwanda. *Journal of medical virology* 2007; **79**(12): 1797-801.
115. Rusine J, Ondoa P, Asiimwe-Kateera B, et al. High Seroprevalence of HBV and HCV Infection in HIV-Infected Adults in Kigali, Rwanda. *PloS one* 2013; **8**(5).
116. Diop-Ndiaye H, Toure-Kane C, Etard JF, et al. Hepatitis B, C seroprevalence and delta viruses in HIV-1 Senegalese patients at HAART initiation (retrospective study). *Journal of medical virology* 2008; **80**(8): 1332-6.
117. Lepretre A, Ba I, Lacombe K, et al. Prevalence and behavioural risks for HIV and HCV infections in a population of drug users of Dakar, Senegal: the ANRS 12243 UDSEN study. *J Int AIDS Soc* 2015; **18**: 19888.
118. Barth RE, Huijgen Q, Tempelman HA, Mudrikova T, Wensing AM, Hoepelman AI. Presence of occult HBV, but near absence of active HBV and HCV infections in people infected with HIV in rural South Africa. *Journal of medical virology* 2011; **83**(6): 929-34.
119. du Plessis R, Webber L, Saayman G. Bloodborne viruses in forensic medical practice in South Africa. *The American journal of forensic medicine and pathology* 1999; **20**(4): 364-8.
120. Gededzha MP, Mphahlele MJ, Lukhwareni A, Selabe SG. Should routine serological screening for HCV be mandatory in HIV/AIDS patients enrolling for HAART in South Africa? *South African medical journal = Suid-Afrikaanse tydskrif vir geneeskunde* 2010; **100**(12): 814-5.
121. Hoffmann CJ, Charalambous S, Thio CL, et al. Hepatotoxicity in an African antiretroviral therapy cohort: The effect of tuberculosis and hepatitis B. *AIDS (London, England)* 2007; **21**(10): 1301-8.
122. Hoffmann CJ, Dayal D, Cheyip M, et al. Prevalence and associations with hepatitis B and hepatitis C infection among HIV-infected adults in South Africa. *International journal of STD & AIDS* 2012; **23**(10): e10-3.
123. Lodenyo H, Schoub B, Ally R, Kairu S, Segal I. Hepatitis B and C virus infections and liver function in AIDS patients at Chris Hani Baragwanath Hospital, Johannesburg. *East African medical journal* 2000; **77**(1): 13-5.
124. Parboosing R, Paruk I, Lalloo UG. Hepatitis C virus seropositivity in a South African Cohort of HIV co-infected, ARV naive patients is associated with renal insufficiency and increased mortality. *Journal of medical virology* 2008; **80**(9): 1530-6.

125. Soni PN, Tait DR, Kenoyer DG, et al. Hepatitis C virus antibodies among risk groups in a South African area endemic for hepatitis B virus. *Journal of medical virology* 1993; **40**(1): 65-8.
126. Mudawi H, Hussein W, Mukhtar M, et al. Overt and occult hepatitis B virus infection in adult Sudanese HIV patients. *International journal of infectious diseases : IJID : official publication of the International Society for Infectious Diseases* 2014; **29**: 65-70.
127. Bowring AL, Luhmann N, Pont S, et al. An urgent need to scale-up injecting drug harm reduction services in Tanzania: prevalence of blood-borne viruses among drug users in Temeke District, Dar-es-Salaam, 2011. *The International journal on drug policy* 2013; **24**(1): 78-81.
128. Franzeck FC, Ngwale R, Msongole B, et al. Viral hepatitis and rapid diagnostic test based screening for HBsAg in HIV-infected patients in rural Tanzania. *PloS one* 2013; **8**(3): e58468.
129. Johnston LG, Holman A, Dahoma M, et al. HIV risk and the overlap of injecting drug use and high-risk sexual behaviours among men who have sex with men in Zanzibar (Unguja), Tanzania. *The International journal on drug policy* 2010; **21**(6): 485-92.
130. Kitundu J, Msengi A, Matee M, et al. Post-transfusion hepatitis C seroprevalence in Tanzanian children. *Annals of tropical paediatrics* 2001; **21**(4): 343-8.
131. Matee MI, Magesa PM, Lyamuya EF. Seroprevalence of human immunodeficiency virus, hepatitis B and C viruses and syphilis infections among blood donors at the Muhimbili National Hospital in Dar es Salaam, Tanzania. *BMC public health* 2006; **6**: 21.
132. Menendez C, Sanchez-Tapias JM, Kahigwa E, et al. Prevalence and mother-to-infant transmission of hepatitis viruses B, C, and E in Southern Tanzania. *Journal of medical virology* 1999; **58**(3): 215-20.
133. Msuya SE, Mbizvo EM, Hussain A, Sam NE, Stray-Pedersen B. Seroprevalence of hepatitis B and C viruses among women of childbearing age in Moshi Urban, Tanzania. *East African medical journal* 2006; **83**(2): 91-4.
134. Mugusi S. Liver enzyme abnormalities and associated risk factors in HIV patients on efavirenz-based HAART with or without tuberculosis co-infection in Tanzania. *Tropical Medicine and International Health* 2012; **17**: 15.
135. Nagu TJ, Bakari M, Matee M. Hepatitis A, B and C viral co-infections among HIV-infected adults presenting for care and treatment at Muhimbili National Hospital in Dar es Salaam, Tanzania. *BMC public health* 2008; **8**: 416.
136. Telatela SP, Matee MI, Munubhi EK. Seroprevalence of hepatitis B and C viral co-infections among children infected with human immunodeficiency virus attending the paediatric HIV care and treatment center at Muhimbili National Hospital in Dar-es-Salaam, Tanzania. *BMC public health* 2007; **7**: 338.
137. Tess BH, Levin A, Brubaker G, et al. Seroprevalence of hepatitis C virus in the general population of northwest Tanzania. *The American journal of tropical medicine and hygiene* 2000; **62**(1): 138-41.
138. Waddell RD, Magesa PM, Pallangyo KJ, et al. Coinfection with HIV and HCV in a blood bank population in Dar es Salaam, Tanzania. *Journal of clinical virology : the official publication of the Pan American Society for Clinical Virology* 2006; **36**(3): 237-8.
139. Muro FJ, Fiorillo SP, Sakasaka P, et al. Seroprevalence of Hepatitis B and C Viruses Among Children in Kilimanjaro Region, Tanzania. *Journal of the Pediatric Infectious Diseases Society* 2013; **2**(4): 320-6.
140. Bessimbaye N, Moussa AM, Mbanga D, et al. Seroprevalence of HBs Ag and of anti-HCV antibodies among HIV infected people in N'Djamena, Chad. [French]

Seroprevalence de l'Ag HBs et de l'anticorps Anti VHC chez les personnes infectees par le VIH1 a N'Djamena, Tchad. *Bulletin de la Societe de pathologie exotique (1990)* 2014; **107**(5): 327-31.
141. Agbodjan E, Prince-David M, Nicot T, Dagnra C, Denis F. [Serologic and genomic research by PCR of hepatitis C virus in different populations in Lome (Togo)]. *Bulletin de la Societe de pathologie exotique (1990)* 1995; **88**(5): 219-24.
142. Kilani B, Ammari L, Marrakchi C, et al. Seroepidemiology of HCV-HIV coinfection in Tunisia. *La Tunisie medicale* 2007; **85**(2): 121-3.

143. Maaref F, Kilani B, Ammari L, et al. [Prevalence of hepatitis G, B and C virus infections among positive HIV population in a Tunisian Hospital, La Rabta, Tunis]. *Pathologie-biologie* 2011; **59**(4): 213-6.
144. Jackson JB, Guay L, Goldfarb J, et al. Hepatitis C virus antibody in HIV-1 infected Ugandan mothers. *Lancet* 1991; **337**(8740): 551.
145. Mullis CE, Laeyendecker O, Reynolds SJ, et al. High frequency of false-positive hepatitis C virus enzyme-linked immunosorbent assay in Rakai, Uganda. *Clinical infectious diseases : an official publication of the Infectious Diseases Society of America* 2013; **57**(12): 1747-50.
146. O'Reilly JI, Ocama P, Opio CK, et al. Risk Factors and Seroprevalence of Hepatitis C among Patients Hospitalized at Mulago Hospital, Uganda. *Journal of tropical medicine* 2011; **2011**: 598341.
147. Seremba E, Ocama P, Opio CK, et al. Poor performance of hepatitis C antibody tests in hospital patients in Uganda. *Journal of medical virology* 2010; **82**(8): 1371-8.
148. Walusansa V, Kagimu M. Screening for hepatitis C among HIV positive patients at Mulago hospital in Uganda. *African health sciences* 2009; **9**(3): 143-6.
149. Baseke J, Musenero M, Mayanja-Kizza H. Prevalence of hepatitis B and C and relationship to liver damage in HIV infected patients attending Joint Clinical Research Centre Clinic (JCRC), Kampala, Uganda. *African health sciences* 2015; **15**(2): 322-7.
150. Kapembwa KC, Goldman JD, Lakhi S, et al. HIV, Hepatitis B, and Hepatitis C in Zambia. *Journal of global infectious diseases* 2011; **3**(3): 269-74.
151. Oshitani H, Kasolo F, Luo NP, et al. Low prevalence of hepatitis C virus infection in Lusaka, Zambia. *Transactions of the Royal Society of Tropical Medicine and Hygiene* 1995; **89**(4): 380.
152. Kallestrup P, Zinyama R, Gomo E, et al. Low prevalence of hepatitis C virus antibodies in HIV-endemic area of Zimbabwe support sexual transmission as the major route of HIV transmission in Africa. *AIDS (London, England)* 2003; **17**(9): 1400-2.
